# Utility of a Smartphone Based System (cvrPhone) to Predict Short-term Arrhythmia Susceptibility

**DOI:** 10.1038/s41598-019-50487-4

**Published:** 2019-10-10

**Authors:** Kwanghyun Sohn, Steven P. Dalvin, Faisal M. Merchant, Kanchan Kulkarni, Furrukh Sana, Shady Abohashem, Jagmeet P. Singh, E. Kevin Heist, Chris Owen, Eric M. Isselbacher, Antonis A. Armoundas

**Affiliations:** 1 Cardiovascular Research Center, Massachusetts General Hospital, Boston, MA USA; 2 Cardiology Division, Emory, University School of Medicine, Atlanta, GA USA; 3 Institute for Medical Engineering and Science, Massachusetts Institute of Technology Cambridge, MA, USA; 4 Cardiology Division, Cardiac Arrhythmia Service, Massachusetts General Hospital, Boston, MA USA; 5 Neurosurgery Division, Massachusetts General Hospital, Boston, MA USA; 6 Healthcare Transformation Lab, Massachusetts General Hospital, Boston, MA USA

**Keywords:** Cardiology, Diagnosis

## Abstract

Repolarization alternans (RA) has been implicated in the pathogenesis of ventricular arrhythmias and sudden cardiac death. We developed a 12-lead, blue-tooth/Smart-Phone (Android) based electrocardiogram (ECG) acquisition and monitoring system (cvrPhone), and an application to estimate RA, in real-time. In *in-vivo* swine studies (N = 17), 12-lead ECG signals were recorded at baseline and following coronary artery occlusion. RA was estimated using the Fast Fourier Transform (FFT) method using a custom developed algorithm in JAVA. Underlying ischemia was detected using a custom developed ischemic index. RA from each lead showed a significant (p < 0.05) increase within 1 min of occlusion compared to baseline (n = 29). Following myocardial infarction, spontaneous ventricular tachycardia episodes (n = 4) were preceded by significant (p < 0.05) increase of RA prior to the onset of the tachy-arrhythmias. Similarly, the ischemic index exhibited a significant increase following myocardial infarction (p < 0.05) and preceding a tachy-arrhythmic event. In conclusion, RA can be effectively estimated using surface lead electrocardiograms by analyzing beat-to-beat variability in ECG morphology using a smartphone based platform. cvrPhone can be used to detect myocardial ischemia and arrhythmia susceptibility using a user-friendly, clinically acceptable, mobile platform.

## Introduction

Electrocardiographic (ECG) alternans, a phenomenon of beat-to-beat oscillation in electrocardiographic waveforms during the repolarization phase of the cardiac cycle also known as repolarization alternans (RA), has been demonstrated to be an important marker of cardiac electrical instability and ventricular tachy-arrhythmic events (VTE)^1,2^. Specifically, the presence of microvolt level RA during low level exercise has been identified as a marker of ventricular arrhythmia susceptibility and can be used to guide implantable cardioverter defibrillator (ICD) therapy in patients with structural heart disease.

However, beyond a risk stratification marker for patients that are candidates to receive ICD therapy, recent clinical studies have also indicated that elevated levels of RA may have important predictive significance of short-term arrhythmia susceptibility. Analysis of body-surface ECG signals from ambulatory patients (Holter monitors) with coronary artery disease has demonstrated a sharp surge in the magnitude of RA within minutes prior to spontaneous VTEs^3^. Analysis of intra-cardiac electrograms (EGMs) from ICDs has demonstrated a sharp elevation in RA magnitude immediately prior to spontaneous ventricular arrhythmias^4,5^. However, a similar surge in RA has not been observed prior to induced VTEs or preceding inappropriate ICD discharges^5,6^. Overall, there is significant evidence to support the notion that a heightened state of RA, measured from intra-cardiac electrodes or body-surface leads, is closely associated with an increased risk to a VTE.

On the other hand, as the average age of the US population increases and chronic conditions are becoming more prevalent, there is a need to improve the effectiveness of disease prevention, to enhance access to healthcare, and to sustain healthy independent living. The increased availability of new technologies and an ever-improving health information technology infrastructure, with >90% of American adults owning a cell phone and 55% having a Smart-Phone^7^, indicates that mobile-health technologies will soon function not only as monitoring devices of the cardiac and respiratory systems^8^, but as essential components in managing patients. Therefore, new, low-cost, easy-to-deploy technologies are needed to meet the clinical need for long-term (>1–2 days) respiratory and cardiac monitoring of the ambulatory patient. The central goal of this study is to investigate the hypothesis that one may develop methods for estimating RA, by recording cardiac electrical activity from the body surface, measuring the beat-to-beat variability in the morphology of ECG waveforms, and using the measured beat-to-beat variability to estimate the RA using the on-board computing power of a Smart-Phone, in order to alert the patient and the treating physician of an impending arrhythmia.

## Methods

### Animal studies

17 male Yorkshire swine (40–45 kg) were anesthetized and instrumented in the Animal Electrophysiology Laboratory of the Massachusetts General Hospital, following previously described methods^9^. Anesthesia was maintained with Isoflurane (1.5–5%), and each animal was intubated and was mechanically ventilated. Ιnvasive blood pressure was monitored through an arterial line.

Briefly, percutaneous vascular access was obtained in the jugular veins and femoral arteries and veins, as previously described, using standard Seldinger techniques^10^. Decapolar catheters were placed in the coronary sinus (CS), right ventricle (RV), right atrium (RA), and left ventricle (LV). An inferior vena cava catheter was inserted as a reference electrode for unipolar signals.

Percutaneous techniques were used to induce coronary artery ischemia, in a closed-chest model^9,11–14^. Briefly, either the mid left circumflex or the mid left anterior descending coronary arteries were occluded with a balloon using standard angioplasty techniques. Ischemia was validated and confirmed by hand injections of contrast into the coronary in which case no-flow, or manifestation of ECG changes were indications of full occlusion. Intravenous unfractionated heparin was administered (4000 units prior to engaging the coronary artery, followed by 1000 units/hour during balloon inflation).

### The hardware architecture

**Figure 1:**
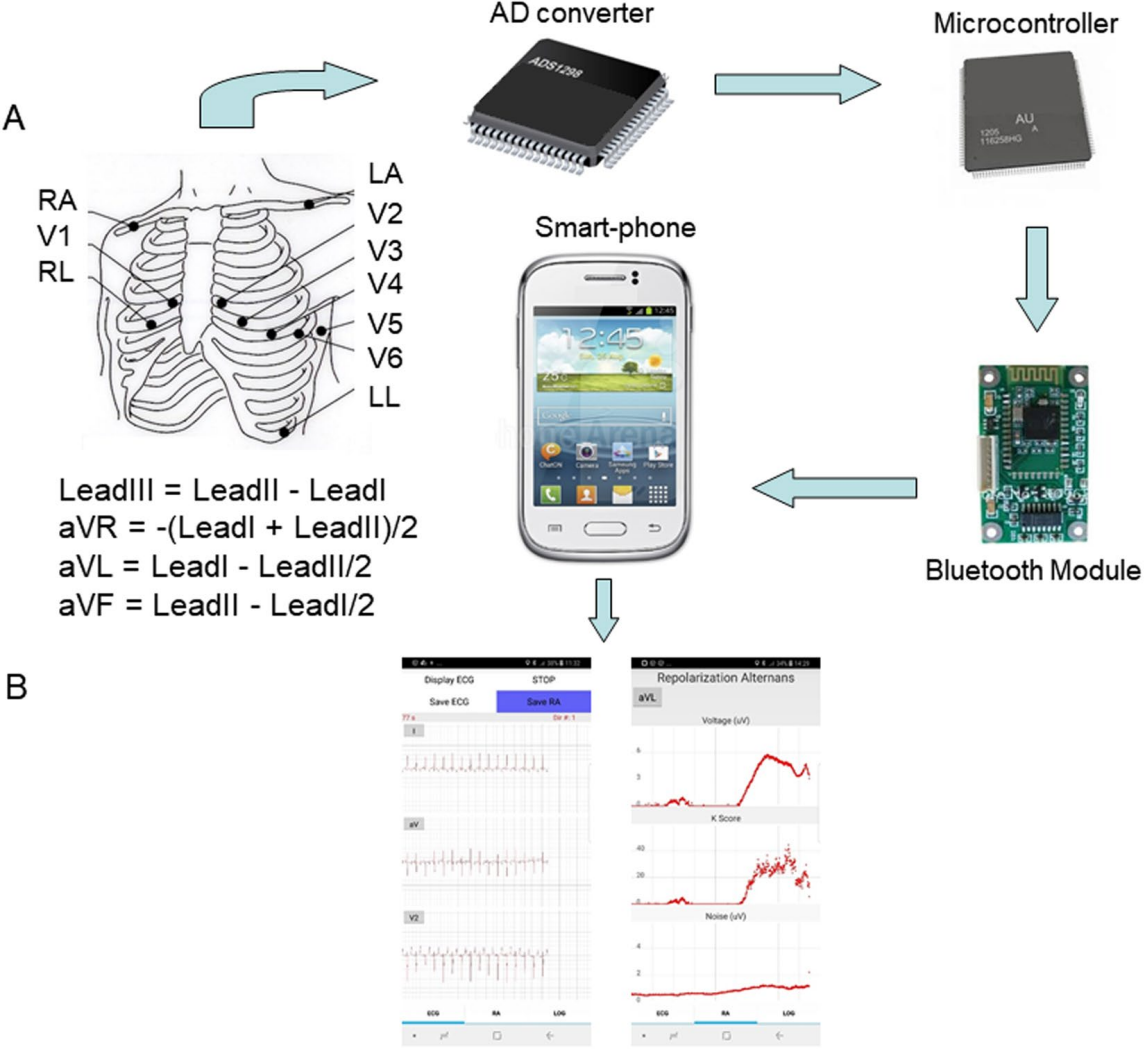
The smartphone-based repolarization alternans monitoring system. The Bluetooth-enabled ECG acquisition device is composed of three parts: An analog-to-digital (AD) converter, a microcontroller board, and a Bluetooth module. The AD converter amplifies and digitizes the signals from the ten electrodes on the torso, and the microcontroller transmits the signals to the smartphone through the Bluetooth module. Then, the smartphone calculates repolarization alternans indices for each lead in real-time.

The hardware architecture of the system has been previously described^8^. Briefly, the ECG device is composed of an analog-to-digital (A/D) converter, a microcontroller board, and a Bluetooth module (Fig. 1A). Following amplification and digitization of the analog ECG signal by the AD converter, they are transmitted by the microcontroller to the smartphone at the user’s request (Fig. 1B). We have validated that signals can be uninterruptedly communicated through the Bluetooth, up to 10 m away from the smartphone, at a baud rate of 115200. The microcontroller was programmed using the open-source, Arduino 1.5.4.

The settings of the AD converter were: sampling rate at 500 samples/s, gain at 12 and reference voltage at 24 V. Reference voltage for the precordial leads was the Wilson Central Terminal defined as RA + LA + LL)/3). Although, the AD converter has 24 bit resolution, that was reduced to 16 bit in order to reduce the transmission load via Bluetooth. The range of the ECG signal is ±12.5 mV, and its resolution is ~0.38 μV.

### Android smartphone application

The application is consisted of three threads: the user-interface, the Bluetooth, and the real-time-calculation. The user is provided with diverse options through the user-interface thread, such as to display the ECG signals and the estimation results. The Bluetooth thread receives the ECG signals from the microcontroller. The real-time-calculation thread estimates RA indices for each lead, independently, and in real-time.

### Body surface ecg data analysis

RA is estimated using a previously described algorithm^2,4,9,15^. Briefly, we first obtain preliminary R-wave detection by applying a software-based QRS detection algorithm to a selected ECG lead. These, preliminary R-wave detections are refined and abnormal beats (i.e. premature ventricular complexes -PVCs- and aberrantly conducted beats) are identified by employing a template-matching QRS alignment algorithm and substituted with a median odd or even template beat (estimated from the odd or even ‘normal’ beats respectively in the 128 beat sequence), depending on whether the abnormal beat is an odd or an even beat^2,9^

Then, repolarization interval boundaries for RA analysis are independently determined for each of the body surface leads, due to variability in the morphology and timing of the T-wave between leads. Briefly, the power method identifies the onset/offset points at time points corresponding to 5% and 95% of the cumulative sum of the signal power^16^, is used for ECG signal waveform annotation.

The, RA is estimated using the spectral method for each 128-beat data sequence (using a 512-point power spectrum to improve the frequency-domain resolution), as previously described^2,9,15,17^. For each lead, spectral analysis is independently performed in order to account for the spatial variability of RA, and RA indices are estimated as follows:

$${\rm{alternans}}\,{\rm{voltage}}\,({\rm{\mu }}V)=\sqrt{{\rm{alternans}}\,{\rm{peak}}-{{\rm{\mu }}}_{{\rm{noise}}}}$$

$${{\rm{K}}}_{{\rm{score}}}=\frac{{\rm{alternans}}\,{\rm{peak}}-{{\rm{\mu }}}_{{\rm{noise}}}}{{{\rm{\sigma }}}_{{\rm{noise}}}}$$

where, the alternans peak is the peak in the aggregate power spectrum corresponding to 0.5 cycles/beat and the mean (µ_noise_) and the standard deviation (σ_noise_) of the alternans noise are estimated in a predefined spectral window (0.43–0.46 cycles/beat) of the power spectrum. The alternans voltage measures directly the presence of RA while the K_score_ is a measure of the statistical significance of the alternans voltage. For each lead, RA is estimated on a beat-by-beat basis using a rolling 128-beat window that is shifted one beat at a time.

### Ischemic index estimation

ST-segment elevation or depression has been well established as a significant marker of MI^18^. We have previously introduced the ischemic index^19^, which is defined as the absolute value of the ratio of ST-height to the QR-amplitude. The ST-height is defined as the mean amplitude of the whole ST-segment above or below the isoelectric baseline, when the polarity at both ends of the ST-segment is the same; if the polarity is different, then the longer segment is selected as the ST-height.

### Assessment of arrhythmia susceptibility

Arrhythmia susceptibility, under varying states of RA, was assessed using programmed ventricular stimulation (PVS)^20^, in which a positive outcome was defined as sustained ventricular tachycardia (VT) or ventricular fibrillation (VF) lasting >30 secs or requiring external defibrillation.

Pacing pulses during PVS were delivered from LV15 and had amplitude and duration 50 mA and 2 msec, respectively. PVS was initiated with a drive train of 8 beats (S1) at a cycle length of 400 milliseconds (ms) with an extra-stimulus (S2) delivered at a coupling interval of approximately 300 ms. The coupling interval for S2 was reduced in 10 ms steps until ventricular refractoriness was reached, at which point S2 was fixed at 20 ms above the point of refractoriness and an S3 was added beginning at a coupling interval 10 ms less than S2. This process was repeated until sustained VT/VF was induced or ventricular refractoriness was reached on S6, in which case PVS was deemed non-inducible under those conditions.

In order to quantify the outcomes of PVS across different RA states, we developed a single “score” rank parameter (S_rank_) which assigned the highest score (highest arrhythmia susceptibility) to the intervention that required (i) the smallest number of extra-stimuli during PVS to induce an arrhythmia, or (ii) if the number of extra-stimuli was the same, to the intervention with the smallest coupling interval between S1 and S_last_, both of which suggest less aggressive stimulation was necessary to induce sustained VT/VF reflecting a more vulnerable arrhythmic substrate. We recognize that there is no single best validated clinical method to assess arrhythmia susceptibility in a fully quantifiable manner. The S_rank_ score was developed not as surrogate of VT/VF (with a binary outcome), but rather as a method to obtain a quantitative relationship between the level of RA and the likelihood of inducing VT/VF.

If sustained VT/VF was induced, biphasic external defibrillation was performed using 150 joules with paddles placed on the chest of the animal and a rest period of ~10 min was allowed after each positive PVS.

### Statistical methods

Aggregate variables are expressed as mean ± standard deviation. Box-plot representation including the median, 90–10% and 75–25% percentiles was used to demonstrate statistical properties of the estimated data sequences. For each RA parameter, a baseline distribution was obtained by collecting the values of that parameter over all time periods before occlusion (t < = 0). Comparisons were then made for each of the alternans noise (µ_noise_), alternans voltage and K_score_, for each lead, between the baseline distribution and the distribution corresponding to each minute after occlusion (t > 0), and a p value was obtained using the Kruskal Wallis test. A threshold value of 0.05 divided by the number of time intervals after occlusion was calculated. Statistical significance at any time interval was then determined based on two factors: (i) the p value resulting from the comparison between the baseline distribution with distribution at that particular interval is less than the threshold value, and (ii) the median of the baseline distribution is less than the median of the distribution at that particular interval. A statistically significant p value is denotted by an “*. Statistical analysis was performed using MATLAB (MathWorks Inc, Natick, MA).

### Ethical approval

The animal studies were approved by the institutional review board and the subcommittee on research animal care at Massachusetts General Hospital. All experiments were performed in accordance with relevant guidelines and regulations.

## Results

### Smartphone-based repolarization alternans estimation

**Figure 2:**
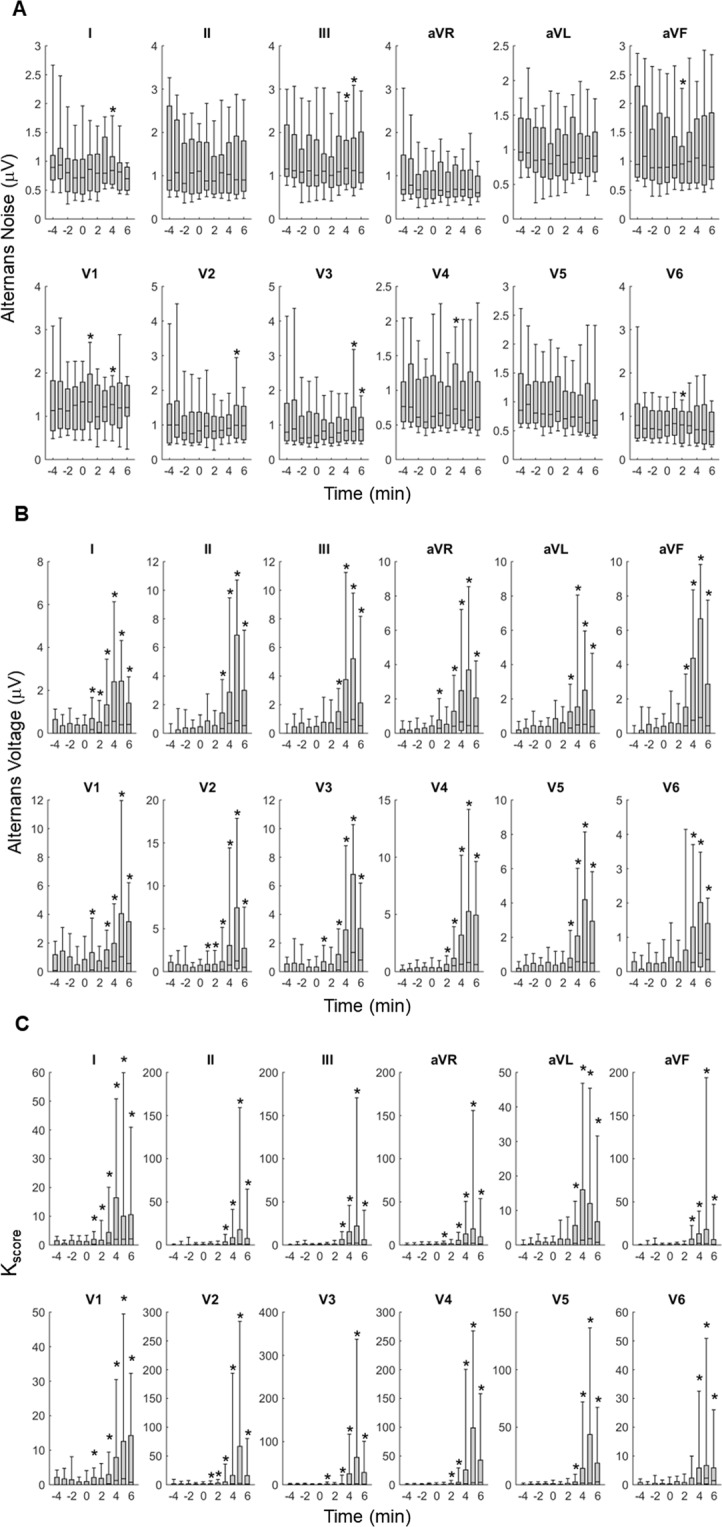
Coronary artery occlusion induced temporal changes of the estimated repolarization alternans (ST-segment and T-wave) indices (n = 29 records; N = 17 animals): (**A**) alternans noise (µ_noise_), (**B**) alternans voltage, and (**C**) K_score_. Time zero indicates the balloon inflation moment. Each bar graph represents 10, 25, 50, 75 and 90 percentiles of the corresponding alternans index values beat-by-beat estimated for all animals for 1 minute time span. Asterisk indicates statistically significant increase after occlusion, compared to before occlusion (p < 0.0001 for the alternans noise, p < 0.0001 for the alternans voltage and p < 0.05 for the K_score_).

In Fig. 2, we observe summary results (n = 29 records, N = 17 animals) of coronary artery occlusion induced temporal changes of the estimated RA (that involves both the ST-segment and T-wave) indices: (A) alternans noise (µ_noise_), (B) alternans voltage, and (C) K_score_. Time zero indicates the timing of the balloon inflation. Across all 12 ECG leads a significant change (p < 0.05) of the alternans noise (in a few leads), voltage and K_score_ after occlusion, compared to before occlusion, is observed.

### Repolarization alternans before a tachy-arrhythmic event

**Figure 3:**
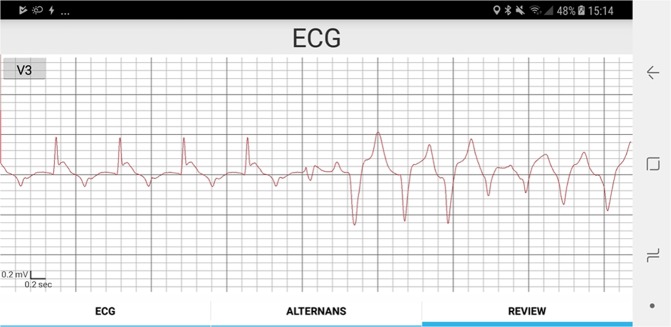
ECG signal (lead V3) displaying spontaneous transition to ventricular tachycardia after coronary artery occlusion.

**Figure 4:**
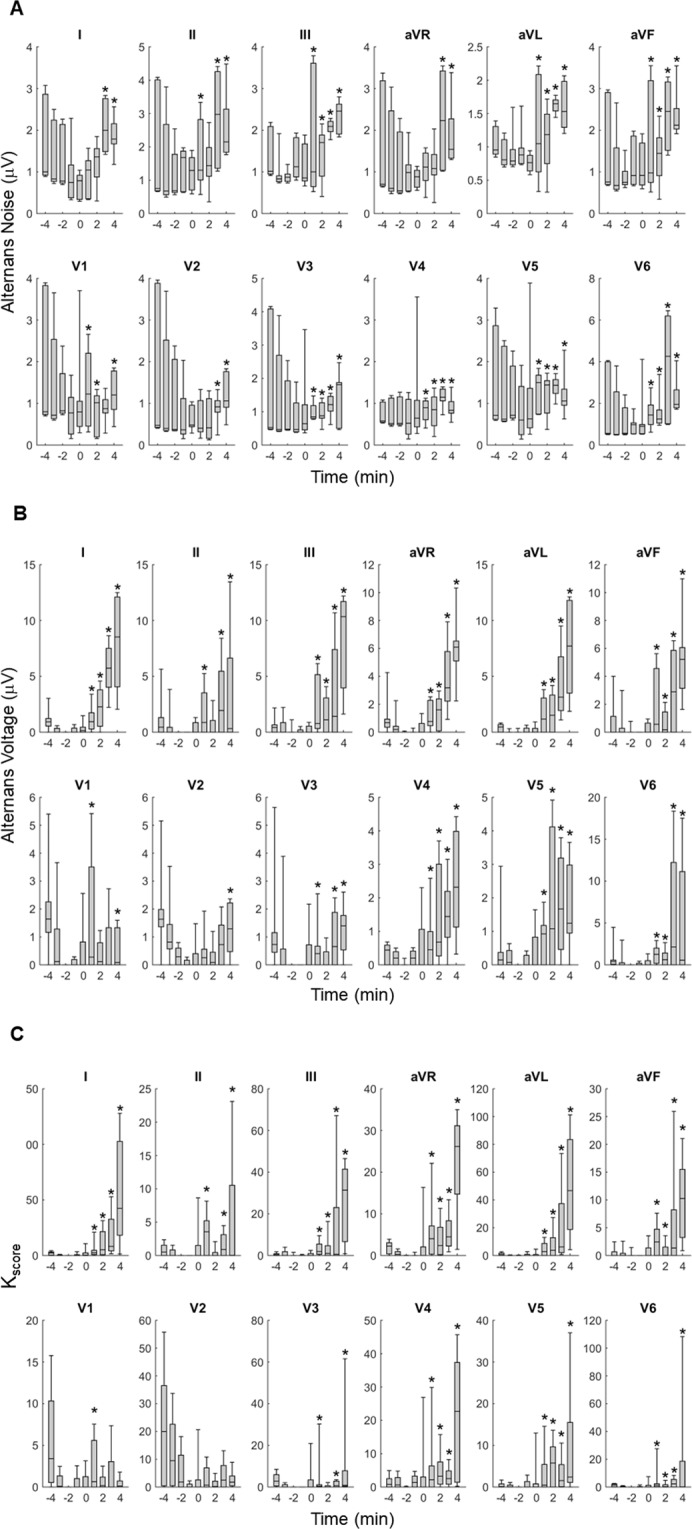
Temporal changes of the repolarization alternans (ST-segment and T-wave) indices during myocardial infarction that led to spontaneous ventricular tachycardia/fibrillation (n = 4 records; 4 animals): (**A**) alternans noise (µ_noise_), (**B**) alternans voltage, and (**C**) K_score_. Time zero indicates the balloon inflation moment. Each bar graph represents 10, 25, 50, 75 and 90 percentiles of the corresponding alternans index estimated on a beat-by-beat basis for all animals, in 1 min time intervals. The asterisk indicates a statistically significant increase after occlusion compared to before occlusion (p < 0.05 for the alternans noise, p < 0.05 for the alternans voltage and p < 0.05 for the K_score_).

In Fig. 3, we present a sample ECG signal (lead V3) during coronary artery occlusion, while the heart-rhythm transitions from sinus to VT. In Fig. 4A–C, we observe summary results of the alternans indices following myocardial infarction, reflecting temporal changes that led led to spontaneous VT/VF (n = 4 records; N = 4 animals): (A) alternans noise (µ_noise_), (B) alternans voltage, and (C) K_score_. Time zero indicates the timing of the balloon inflation. We observe that the alternans noise level was statistically different (p < 0.05) before compared to after occlusion, and also ischemia led to a statistically significant increase of the alternans voltage (p < 0.05) and K_score_ (p < 0.05) after occlusion, compared to before occlusion.

We compared distributions of alternans noise (µ_noise_), alternans voltage, and K_score_, between records that exhibited VT/VF (n = 4) and those that did not (n = 25), following myocardial infarction (at times: 0, 1, 2, 3 and 4 min), and we report the obtained range of p-values, resulting from this comparison, in Table 1.

**Table 1:** Range of p-values resulting from comparing distributions of alternans noise, alternans voltage, and K_score_, between records that exhibited VT/VF (n = 4) and those that did not (n = 25), following myocardial infarction (at times: 0, 1, 2, 3 and 4 min, in Figs 2 and 3).

Lead	Alternans Noise	Alternans Voltage	K_score_
I	0 < P < 0.001	0 < P < 0.001	0 < P < 0.001
II	0 < P < 0.001	0.001 < P < 0.733	0.001 < P < 0.257
III	0 < P < 0.186	0.001 < P < 0.843	0.001 < P < 0.068
AVR	0 < P < 0.001	0.001 < P < 0.492	0.001 < P < 0.088
AVL	0 < P < 0.015	0.001 < P < 0.362	0.001 < P < 0.776
AVF	0.001 < P < 0.090	0.001 < P < 0.429	0.001 < P < 0.007
V1	0.001 < P < 0.003	0.001 < P < 0.777	0.001 < P < 0.944
V2	0.001 < P < 0.423	0.001 < P < 0.318	0.001 < P < 0.783
V3	0.001 < P < 0.124	0.001 < P < 0.186	0.001 < P < 0.418
V4	0.001 < P < 0.098	0 < P < 0.001	0 < P < 0.001
V5	0.001 < P < 0.007	0.001 < P < 0.754	0.001 < P < 0.713
V6	0 < P < 0.001	0 < P < 0.001	0.001 < P < 0.243

**Figure 5:**
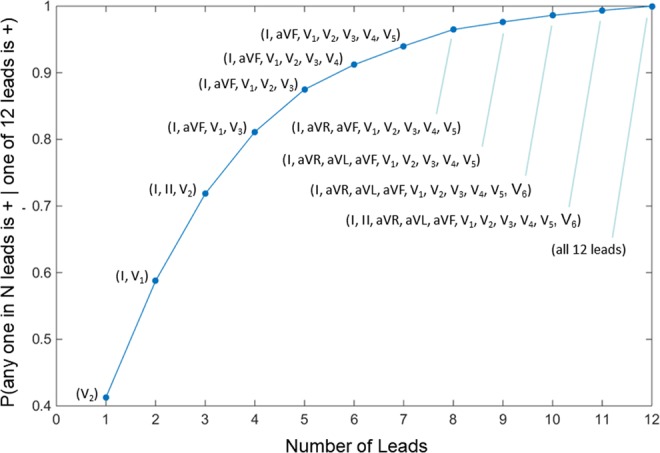
Sensitivity of the 12 lead ECG in detecting RA, that is P(any one in N leads is +| one of 12 leads is +). In the plot one observes the highest performing lead combinations of N leads, for any number of leads ranging from one to twelve. +: indicates positive.

To examine the sensitivity of the 12 lead system in detecting RA we calculated the conditional probability that any one lead in a combination of N leads is positive, given that at least one lead out of all 12 leads is positive: P(any one in N leads is positive | one of 12 leads is positive). We define as positive RA an estimate that satisfies the following criteria: (i) alternans voltage is higher than 0.55 μV, and (ii) K_score_ is higher than 3^9^. If at any instance, we find that any one of the 12 leads is positive, we evaluate if positive alternans can be detected with a combination of N leads, with N ranging from one to twelve. All combinations of N leads out of 12 have been considered for this purpose. Then, the probability for a specific combination of leads is calculated by the ratio between the number of times a positive detection was made to the total number of positive detections by the 12 leads. Once the probabilities are computed over all combinations of size N across all 29 recordings, the average probability over the 29 recordings for each combination was calculated, and the combination yielding the maximum probability for a specific number of leads was reported (Fig. 5). We observe that four leads provide higher than 80% probability that RA is detected and that number raised to more than 90% with six leads.

### Repolarization alternans burden

**Figure 6:**
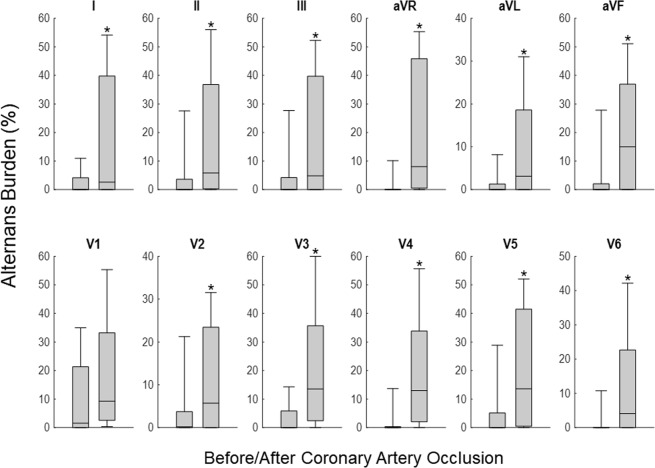
Repolarization alternans (ST-segment and T-wave, RA) burden before and after coronary artery occlusion. RA positive, criteria were defined as: (i) alternans voltage is greater than 0.55 μV, and (ii) K_score_ greater than 3. The RA burden is evaluated on a beat-by-beat basis as a percent of sequences that exhibited significant RA, and percentages of RA incidence are calculated before and after the occlusion separately, for each record. Each bar graph represents 10, 25, 50, 75 and 90 percentiles of alternans burden of all records. An asterisk indicates statistically significant (p < 0.05) difference between the two alternans percents before and after occlusion (n = 29 records; N = 17 animals).

In Fig. 6, we present the alternans burden (%) before and after coronary artery occlusion during MI (n = 29 records; N = 17 animals). Again, we define as positive an RA an estimate that satisfies the criteria above for (i) alternans voltage is higher than 0.55 μV, and (ii) K_score_ is higher than 3^9^.

The incidence of RA is evaluated on a beat-by-beat basis, and the RA burden is evaluated as a percent of sequences that exhibit significant RA; the RA burden is estimated separately after the occlusion, for each record.

We observe that during MI the RA burden is significantly higher (p < 0.05, using the paired T-test), compared to baseline.

### Relationship of ischemic index and repolarization alternans

**Figure 7:**
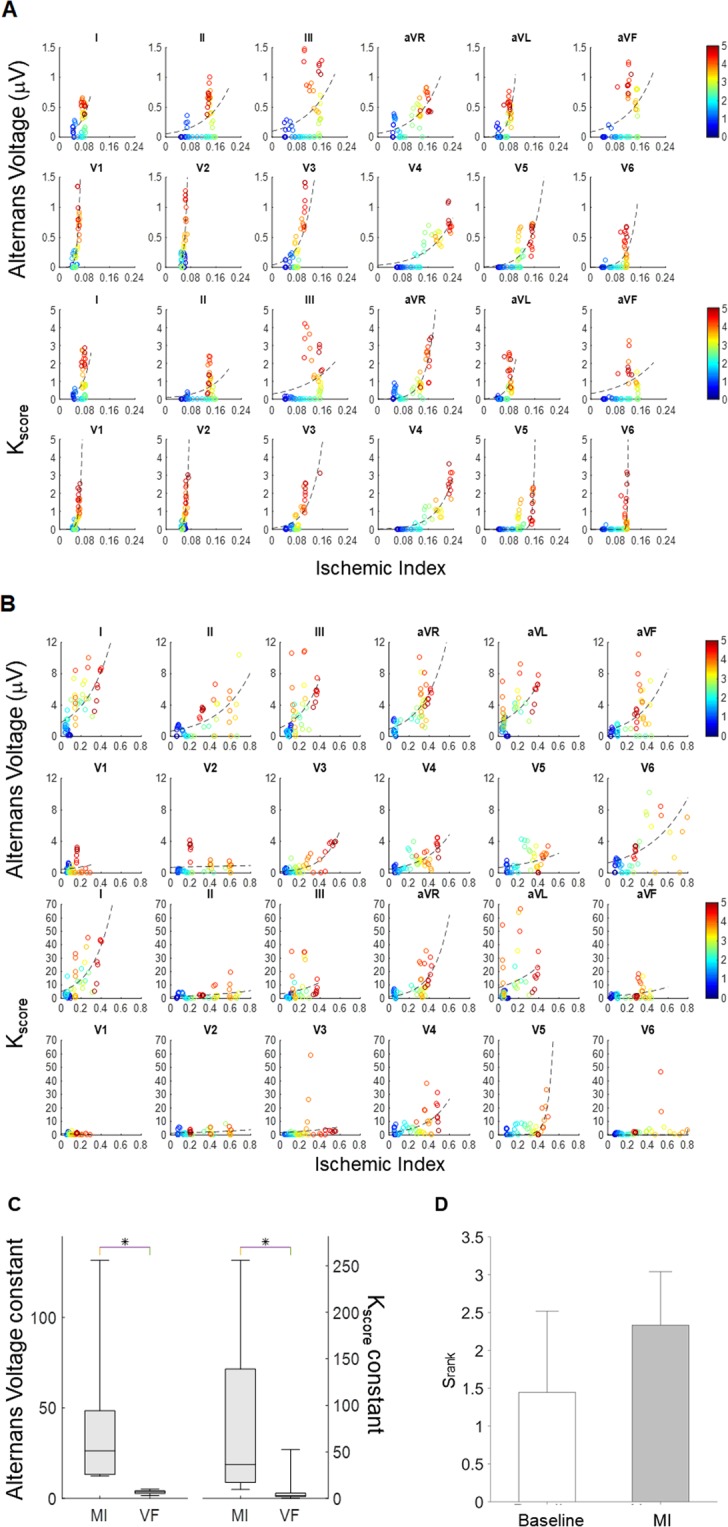
Relationship of repolarization (ST-segment and T-wave) alternans vs ischemic index (**A**) during myocardial infarction and (**B**) preceding ventricular tachycardia/fibrillation. Alternans voltage (μV) versus ischemic index (upper panel), and K_score_ versus ischemic index (lower panel). The color bars on the right side show time after coronary artery occlusion from 0 min to 5 min. The dashed line at each plot represents a data fitting line with a single-term exponential model. (**D**) PVS that was employed at baseline and after coronary artery occlusion, MI (N = 9), to assess the arrhythmogenic potential of RA. Although the S_rank_ at baseline and after coronary artery occlusion was not statistically different, yet it trended towards a higher value after occlusion.

Next, we sought to explore the relationship of RA vs the ischemic index during MI (Fig. 7A) and preceding VT/VF (Fig. 7B). In each figure, the alternans voltage (μV) versus ischemic index is presented in the upper panel, and the K_score_ versus ischemic index, is presented in the lower panel. The color bars on the right side indicate the time after coronary artery occlusion from 0 min to 5 min. The dashed line in each plot represents a data fitting line with a single-term exponential model.

In Fig. 7C, we observe that for both the alternans voltage (p < 0.05) and K_score_ (p < 0.05, using the paired t-test) the constant of the exponential model is significantly smaller before VT/VF, indicating that RA manifests a profound arrhythmogenic substrate.

### RA and arrhythmia susceptibility

To assess the arrhythmogenic potential of RA we employed PVS that was performed at baseline and after coronary artery occlusion (N = 9).

We observed that the S_rank_ at baseline and after coronary artery occlusion was not statistically different (Fig. 7D), yet it trended towards a higher value after occlusion associating RA with a higher arrhythmogenic risk.

## Discussion

In this study, we have shown that RA can be effectively estimated from body surface ECG signals, through Bluetooth, using a smartphone; *second*, the smartphone can provide a viable platform to process ECG signals in real-time and, if needed, enable generation of alerts for the patient and the treating physician of an impending arrhythmia while the patient maintains an ambulatory status; *third*, there is a strong connection between RA and the ischemic index, especially before a tachy-arrhythmic event, indicating the significance of RA in predicting a tachy-arrhythmic event, at least in this model.

Optical mapping studies in normal hearts have shown that discordant (reflecting two areas in the heart that oscillate with opposing phase) APD alternans is linked to a state of reduced cardiac electrical stability, manifested by the observation that when alternans is followed by VF, it only occurs after discordant APD alternans, but never concordant APD alternans^21^.

RA estimated in Holter ECG signals in ambulatory patients with coronary artery disease has shown a marked surge in RA magnitude within minutes preceding a spontaneous VTE^3^. T-wave alternans (TWA) amplitude reached a peak about 10 min prior to the onset of a VTE. Sharp surges in TWA immediately preceding spontaneous VTEs have also been documented in body-surface ECGs in patients hospitalized for acute heart failure^22^; TWA increased from a baseline during 15–30 mins prior to the onset of the VTE and remained elevated until the occurrence of VTE. RA estimated in intra-cardiac EGMs from ICDs has shown a sharp surge prior to spontaneous VTEs^4,5^; however, a similar RA surge has not been noticed prior to induced VTEs or prior to inappropriate ICD shocks^5^. Recently a prospective study in patients with ICDs has confirmed these findings^6^; specifically, the magnitude of T-wave alternans/variability (TWA/V) prior to spontaneous VTE was significantly higher than during any of the control segments, while logistic regression analysis has shown that each 10 μV increase in TWA/V was associated with a 2.2 odds increase of developing a VTE. These observations establish a close temporal relationship between surges in TWA/V and the onset of spontaneous VTEs.

On the other hand, the ischemic index, that quantifies beat-to-beat changes observed in both ventricular depolarization and repolarization during ischemia, provides a personalized, lead-independent measure that accounts for both depolarization^23,24^ and repolarization^25–27^ changes observed during MI. In this study, as well as in prior studies^28^, we have seen that despite the dynamic beat-to-beat and subject-to-subject variability of ECG morphology, the ischemic index presents high stability as well as very low intra- and inter-subject variability under baseline (non-ischemic) conditions^28^, while it exhibits great spatial sensitivity in detecting MI-induced changes and has been linked to VTEs^28^.

In summary, although the magnitude of RA increases in body-surface leads is smaller than that measured in intra-cardiac EGMs^9^, simultaneous measurement of RA from body-surface and intra-cardiac EGMs by our group^9^ and others^29^ has shown a high degree of correlation suggesting that these measurements are reflecting the same electrical phenomenon. The data presented in this study as well as by others support the idea that a sharp increase of RA prior to the onset of spontaneous VTE can be measured from body-surface electrodes and may be used to predict acute arrhythmia susceptibility. In such scenario, a heightened state of the ischemic index and/or RA (compared to that subject’s baseline levels, personalized health care) could serve as a warning and indication that the subject should adopt behavioral changes (i.e. stop exercising) or take medication (i.e. a b-blocker), or seek medical attention.

## Supplementary information

Supplementary Information

## Data Availability

The data will be available to any investigator upon request.
