# Supplementary material for: Utility of a Smartphone Based System (cvrPhone) to Predict Short-term Arrhythmia Susceptibility
Source: Sci Rep. 2019 Oct 10;9:14497. doi: 10.1038/s41598-019-50487-4 (PMC6787075; doi:10.1038/s41598-019-50487-4)

Utility of a Smartphone Based System (cvrPhone) to Predict Short-term Arrhythmia Susceptibility

Kwanghyun Sohn, PhD, Steven P Dalvin, MD, Faisal M. Merchant, MD, Kanchan Kulkarni, PhD, Furrukh Sana, PhD, Shady Abohashem, MD, Jagmeet P. Singh, MD, PhD, E. Kevin Heist, MD, PhD, Chris Owen, MS, Eric M. Isselbacher, MD, MSc

Antonis A. Armoundas, PhD

Hardware Architecture

The ECG device is composed of an analog-to-digital (A/D) converter (ADS1298, Texas Instruments, Dallas, TX), a microcontroller board (Arduino Due AT91SAM3X8E, Atmel, San Jose, CA), and a Bluetooth module (HC-05, Guangzhou HC Information Technology Co., Ltd., Guangzhou, China). The AD converter amplifies and digitizes the analog ECG signal from electrodes, and the microcontroller transmits the digitized ECG signal to the smartphone. Uninterrupted Bluetooth communication could be achieved up to 10 meters away from the smartphone.

Microcontroller Software

The microcontroller receives digitized ECG signals from the AD converter and transmits them to the smartphone through Bluetooth at the user’s request. We used the open-source integrated development environment (IDE) Arduino 1.5.4 for the microcontroller programming. There are two main steps in the function of the embedded software: *first*, initialize the AD converter and the Bluetooth module; and *second*, transmit the ECG signals upon user’s request (Online Supplement Figures S1-S2).

The settings of sampling rate, gain and reference voltage of the AD converter are 500 samples per second (SPS), 12 and 24 V respectively. The Wilson Central Terminal (WCT, (RA+LA+LL)/3) is used as the reference voltage for the precordial leads. The signal from the AD converter has 24 bit resolution, but it is reduced to 16 bit by dropping the upper and lower 4 bits

to reduce the transmission load via Bluetooth. The ECG signal covers ±12.5 mV with resolution of ~0.38 μV. The baud rate of the Bluetooth module is 115200.

After the initialization, the microcontroller repeats sending ECG signals according to the user's "actions." There are three different actions: "save," "display." and "stop": (i) At "save" action, the smartphone displays ECG data on the screen, saves the ECG data, and calculates the ischemic index, RR and TV in real-time. The microcontroller transmits the ECG signals at 500 SPS to the smartphone for the "save" action. (ii) At "display" action, the smartphone just displays the ECG signals on the screen, and the microcontroller transmits every 5th sample of the ECG signals in order to reduce the transmission load. This 100 SPS is enough for the display on the smartphone screen. (iii) At "stop" action, there is no signal transmission.

Online Supplement Figure Captions

Online Supplement Figure 1 Coronary artery occlusion induced temporal changes of the estimated ST-segment alternans indices (n=29 records; N= 17 animals): (A) alternans noise (μ_{noise}), (B) alternans voltage, and (C) K_{score}. Time zero indicates the balloon inflation moment. Each bar graph represents 10, 25, 50, 75 and 90 percentiles of the corresponding alternans index values beat-by-beat estimated for all animals for 1 minute time span. Asterisk indicates statistically significant ($p<0.05$) increase after occlusion, compared to before occlusion ($p<0.0001$ for the alternans noise, $p<0.0001$ for the alternans voltage and $p<0.05$ for the K_{score}).

Online Supplement Figure 2 Coronary artery occlusion induced temporal changes of the estimated T-wave alternans indices (n=29 records; N= 17 animals): (A) alternans noise (μ_{noise}), (B) alternans voltage, and (C) K_{score}. Time zero indicates the balloon inflation moment. Each bar graph represents 10, 25, 50, 75 and 90 percentiles of the corresponding alternans index values beat-by-beat estimated for all animals for 1 minute time span. Asterisk indicates statistically significant ($p<0.05$) increase after occlusion, compared to before occlusion ($p<0.0001$ for the alternans noise, $p<0.0001$ for the alternans voltage and $p<0.05$ for the K_{score}).

Online Supplement Figure 3 Coronary artery occlusion induced temporal changes of the estimated ST-segment alternans indices prior to ventricular tachycardia/fibrillations (n=4 records; N= 4 animals): (A) alternans noise (μ_{noise}), (B) alternans voltage, and (C) K_{score}. Time zero indicates the balloon inflation moment. Each bar graph represents 10, 25, 50, 75 and 90 percentiles of the corresponding alternans index values beat-by-beat estimated for all animals for 1 minute time span. Asterisk indicates statistically significant ($p<0.05$) increase after occlusion, compared to before occlusion ($p<0.05$ for the alternans noise, $p<0.05$ for the alternans voltage and $p<0.05$ for the K_{score}).

Online Supplement Figure 4 Coronary artery occlusion induced temporal changes of the estimated T-wave alternans indices prior to ventricular tachycardia/fibrillations (n=4 records; N= 4 animals): (A) alternans noise (μ_{noise}), (B) alternans voltage, and (C) K_{score}. Time zero indicates the balloon inflation moment. Each bar graph represents 10, 25, 50, 75 and 90 percentiles of the

corresponding alternans index values beat-by-beat estimated for all animals for 1 minute time span. Asterisk indicates statistically significant ($p<0.05$) increase after occlusion, compared to before occlusion ($p<0.0001$ for the alternans noise, $p<0.05$ for the alternans voltage and $p<0.05$ for the K_{score})..

Online Supplement Figure 5 ST-segment alternans burden (A) before and after coronary artery occlusion (n=29 records; N=17 animals) and (B) before and after coronary artery occlusion before ventricular tachycardia/fibrillation (n=4 records; N=4 animals). RA positive, criteria were defined as: (i) alternans voltage is greater than 1 μV, and (ii) K_{score} greater than 3. The RA burden is evaluated on a beat-by-beat basis as a percent of sequences that exhibited significant RA, and percentages of RA incidence are calculated before and after the occlusion separately, for each record. Each bar graph represents 10, 25, 50, 75 and 90 percentiles of alternans burden of all records. An asterisk indicates statistically significant ($p<0.05$) difference of the alternans burden before and after occlusion. Alternans burden (%) with all myocardial infarction tests ($p<0.05$ in (A) and p=NS in (B)).

Online Supplement Figure 6 T-wave alternans burden (A) before and after coronary artery occlusion (n=29 records; N=17 animals) and (B) before and after coronary artery occlusion before ventricular tachycardia/fibrillation (n=4 records; N=4 animals). RA positive, criteria were defined as: (i) alternans voltage is greater than 1 μV, and (ii) K_{score} greater than 3. The RA burden is evaluated on a beat-by-beat basis as a percent of sequences that exhibited significant RA, and percentages of RA incidence are calculated before and after the occlusion separately, for each record. Each bar graph represents 10, 25, 50, 75 and 90 percentiles of alternans burden of all records. An asterisk indicates statistically significant ($p<0.05$) difference between the two alternans percents before and after occlusion. Alternans burden (%) with all myocardial infarction tests (p=<0.05 in (A), p=NS in (B)).

Online Supplement Figure 7 ECG signals displaying spontaneous transition to ventricular tachycardia after coronary artery occlusion: (A) Lead I, (B) Lead II, (C) Lead III, (D) aVR, (E) aVL, (F) aVF, (G) VI, (H) V2, (I) V3, (J) V4, (K) V5, (L) V6.

Online Supplement Figure 1

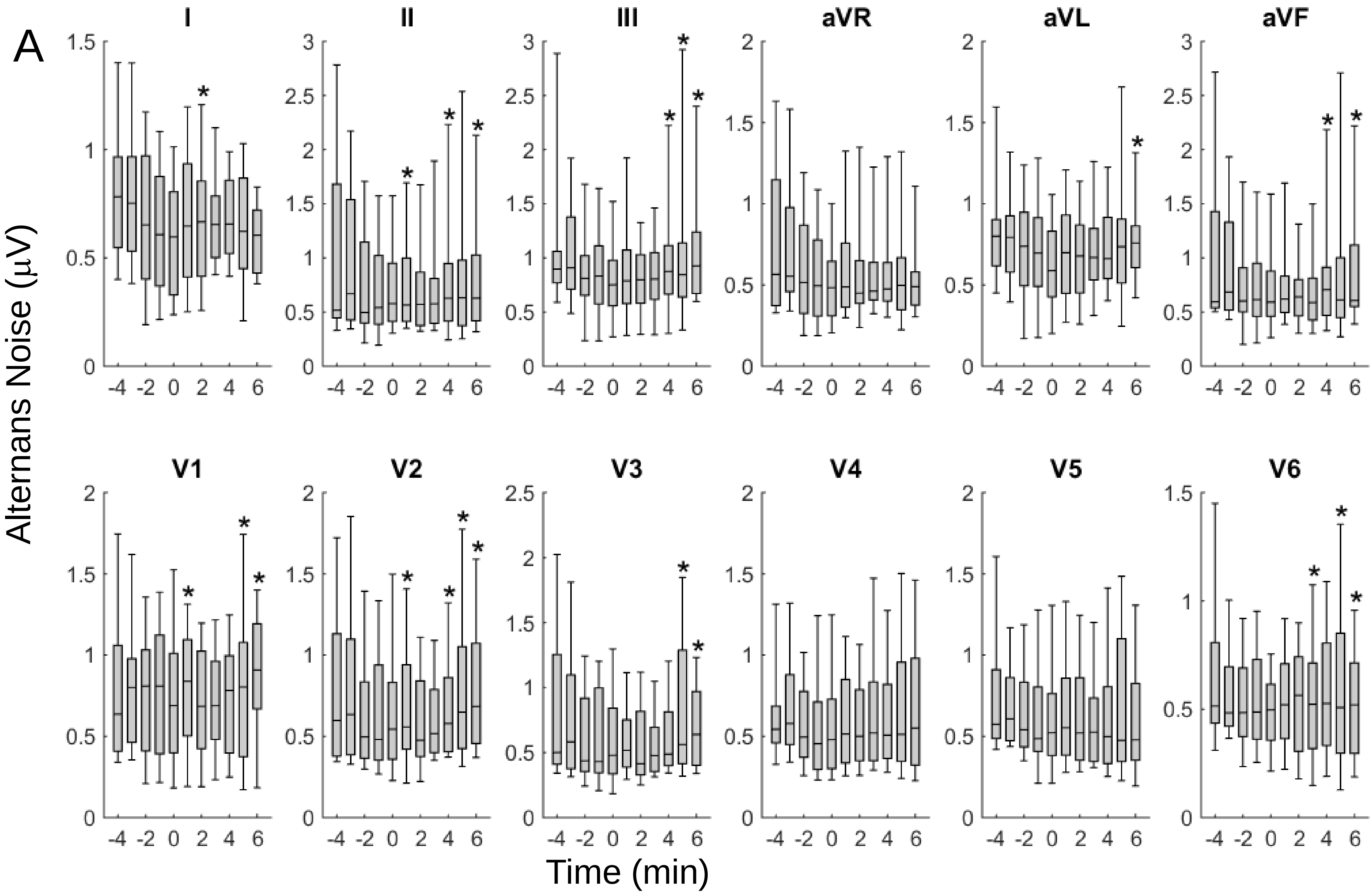

Online Supplement Figure 1

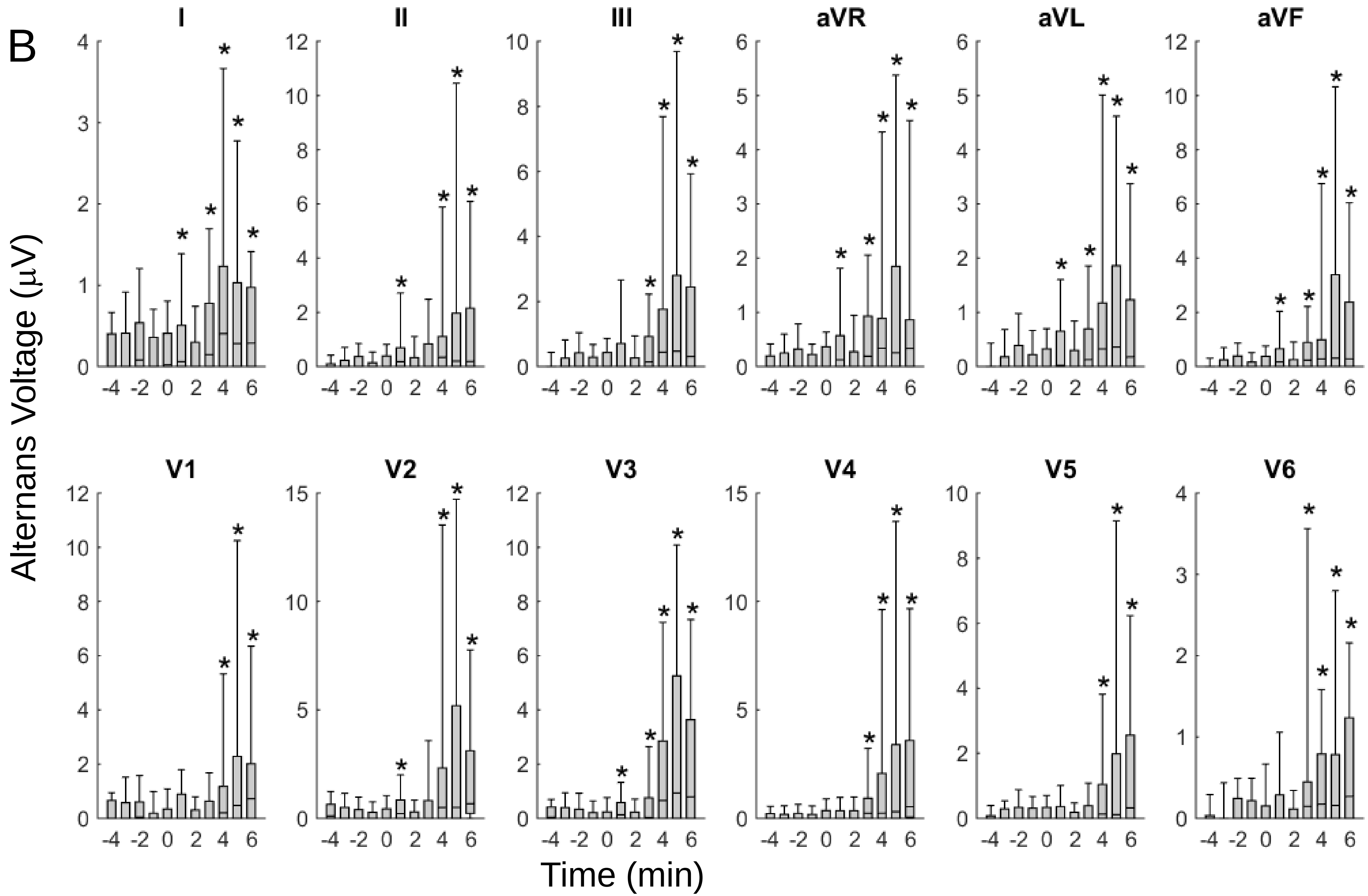

Online Supplement Figure 1

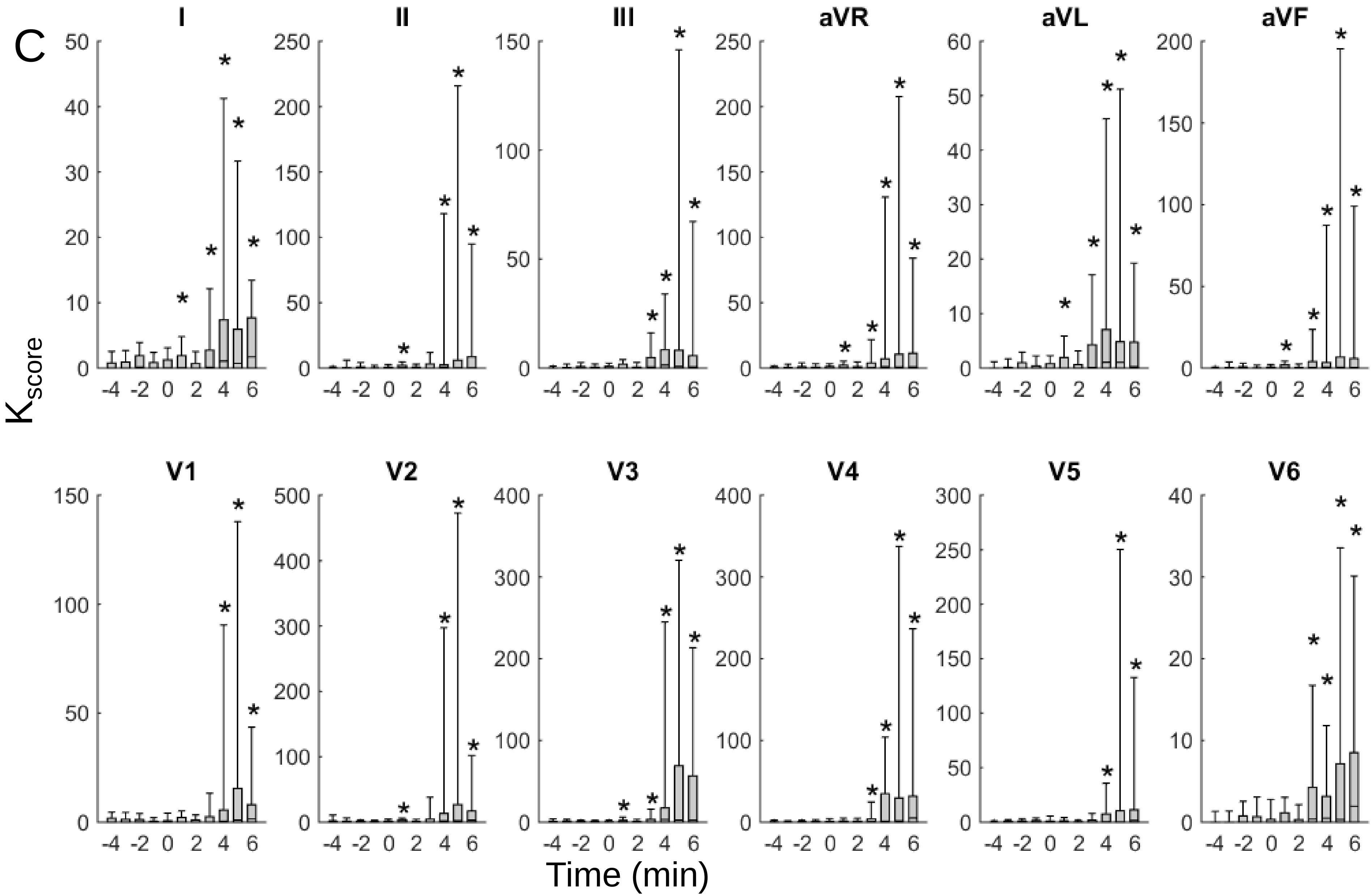

Online Supplement Figure 2

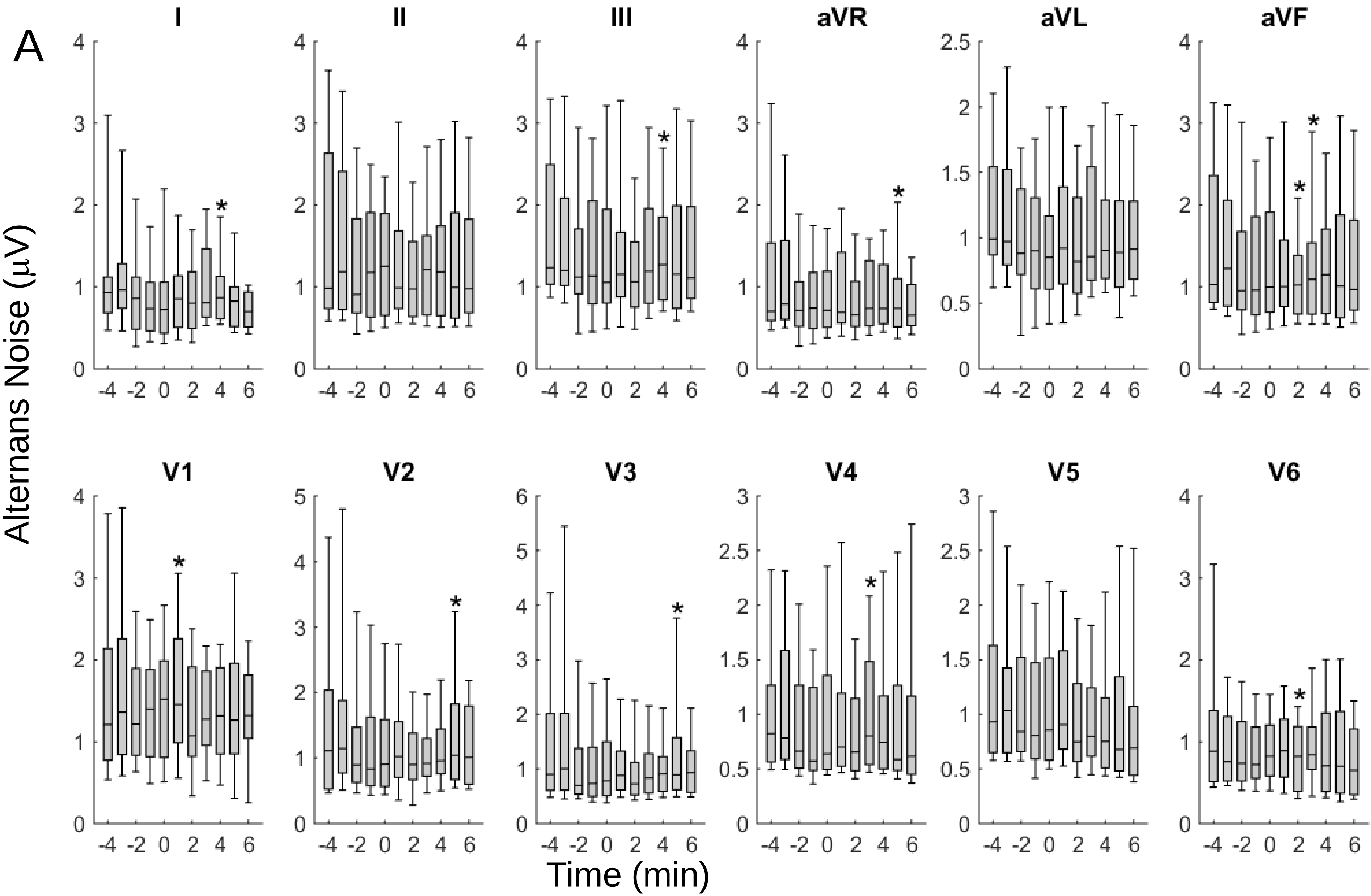

Online Supplement Figure 2

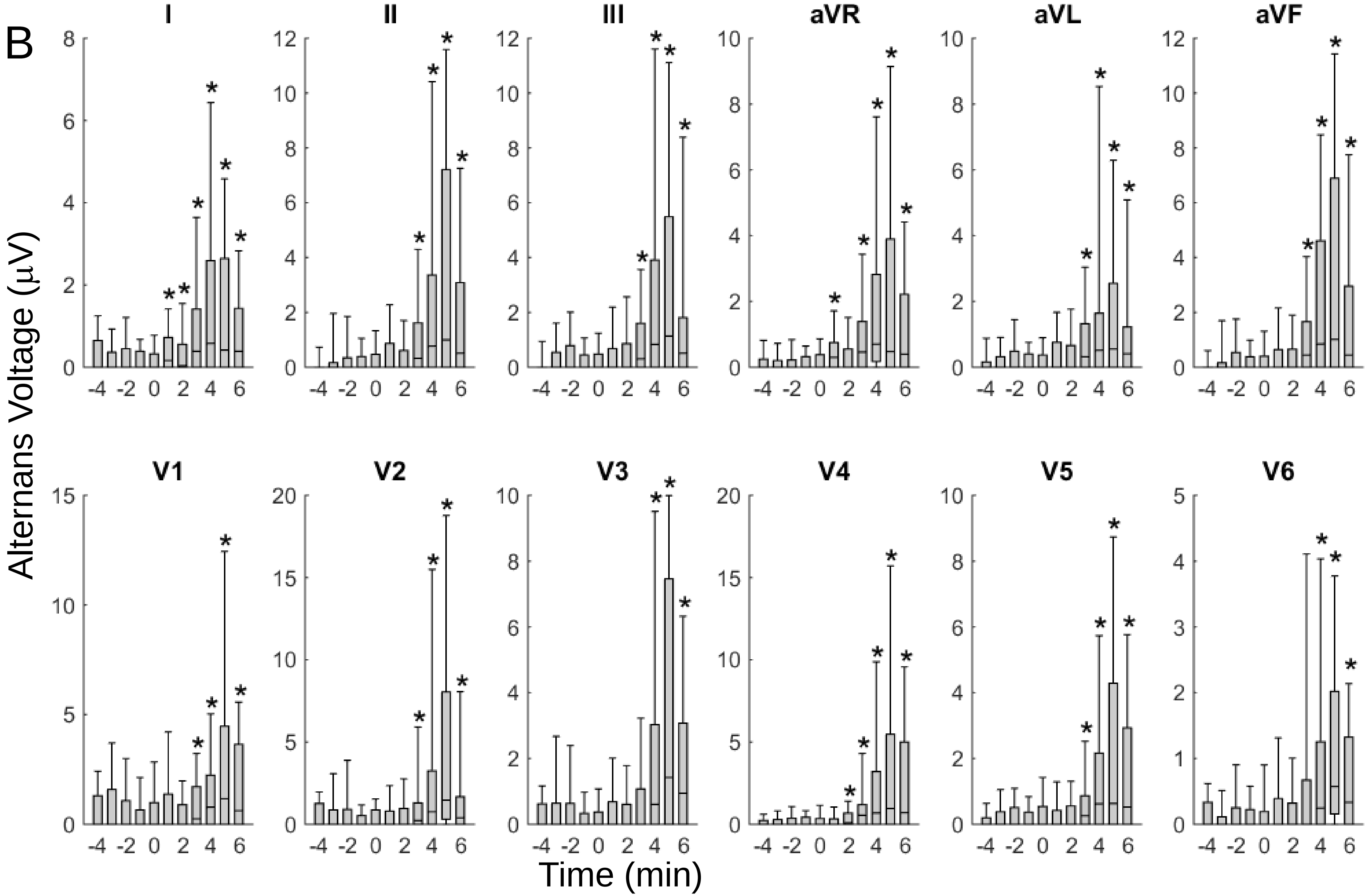

Online Supplement Figure 2

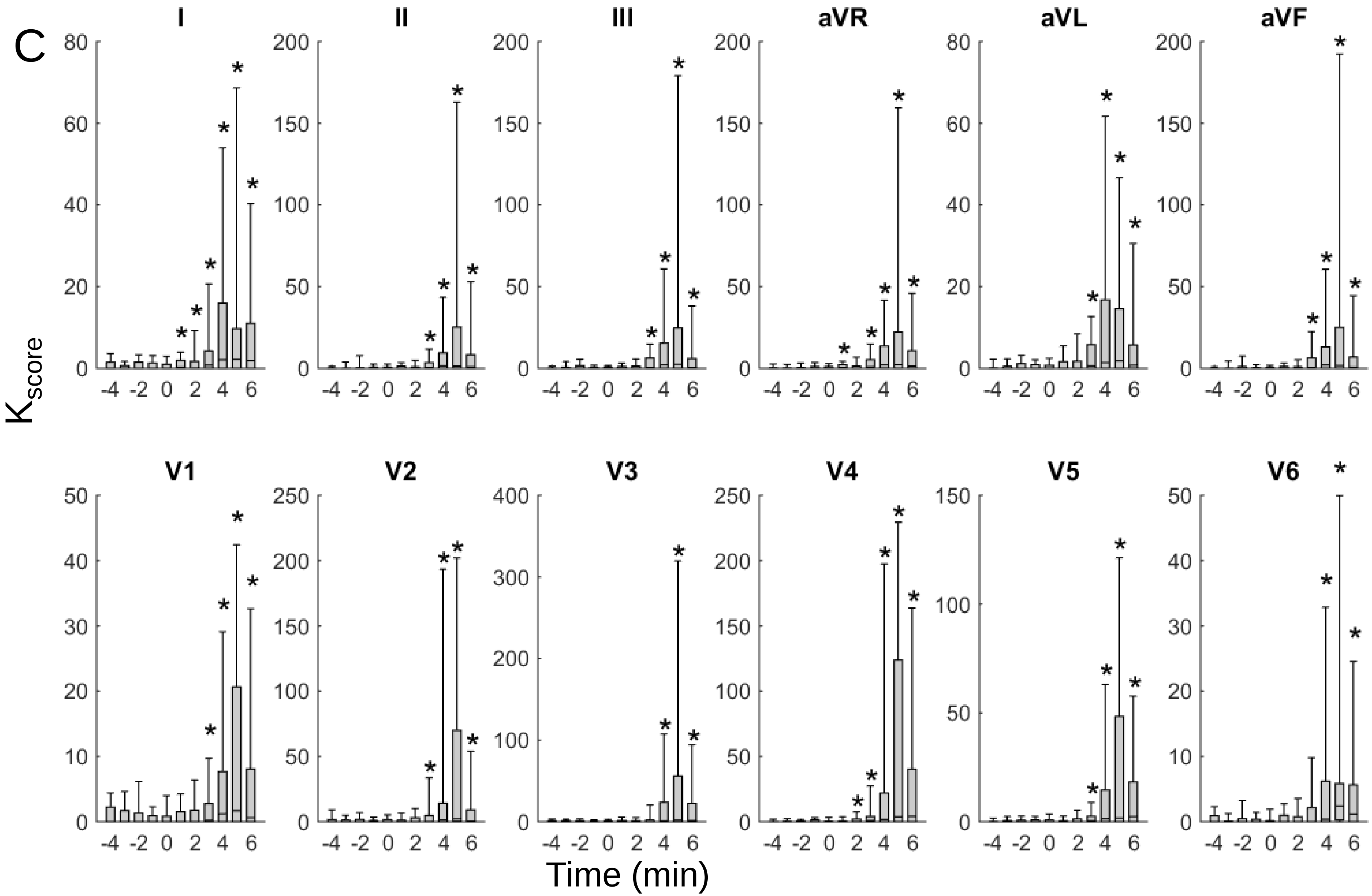

Online Supplement Figure 3

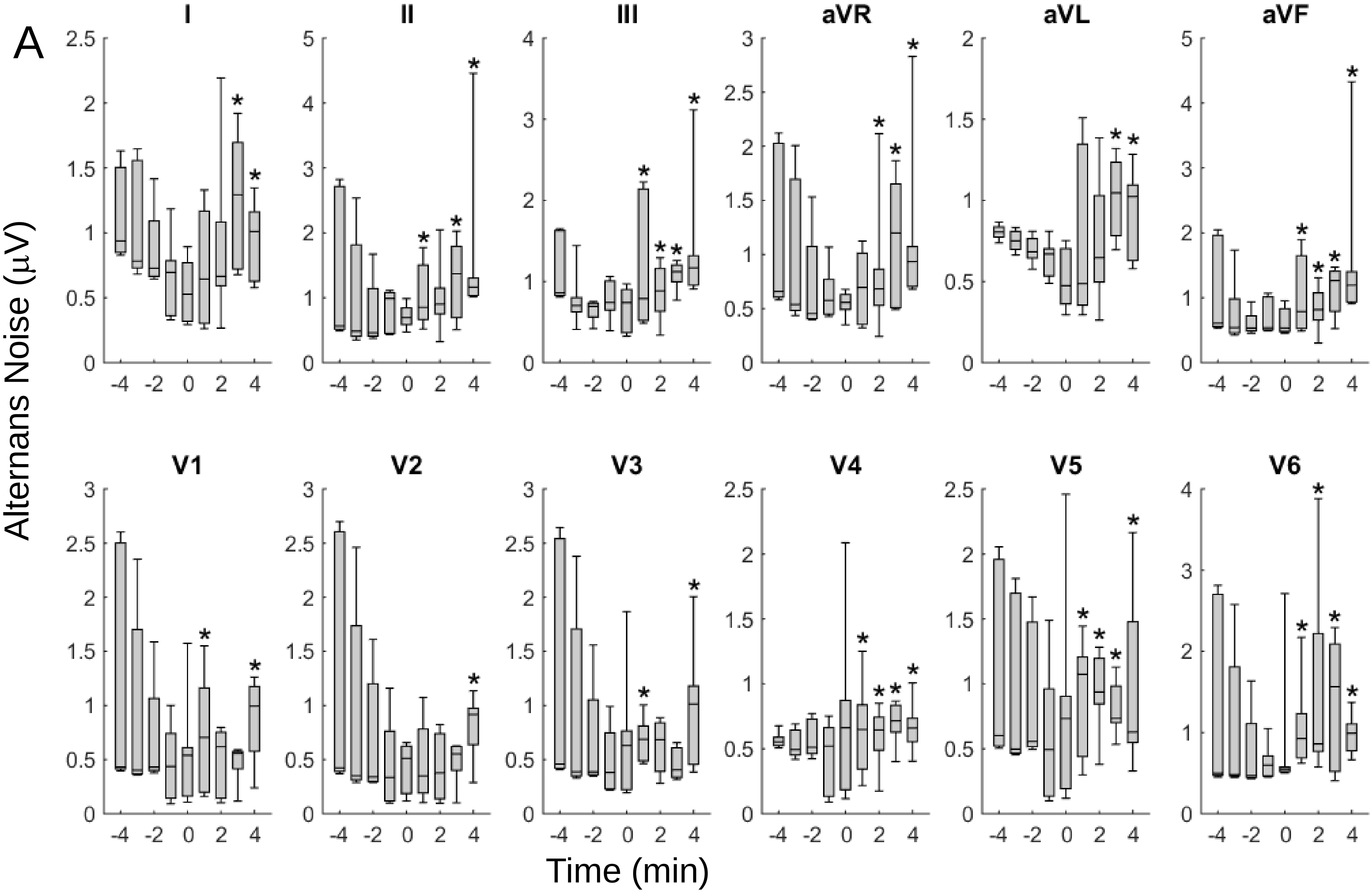

Online Supplement Figure 3

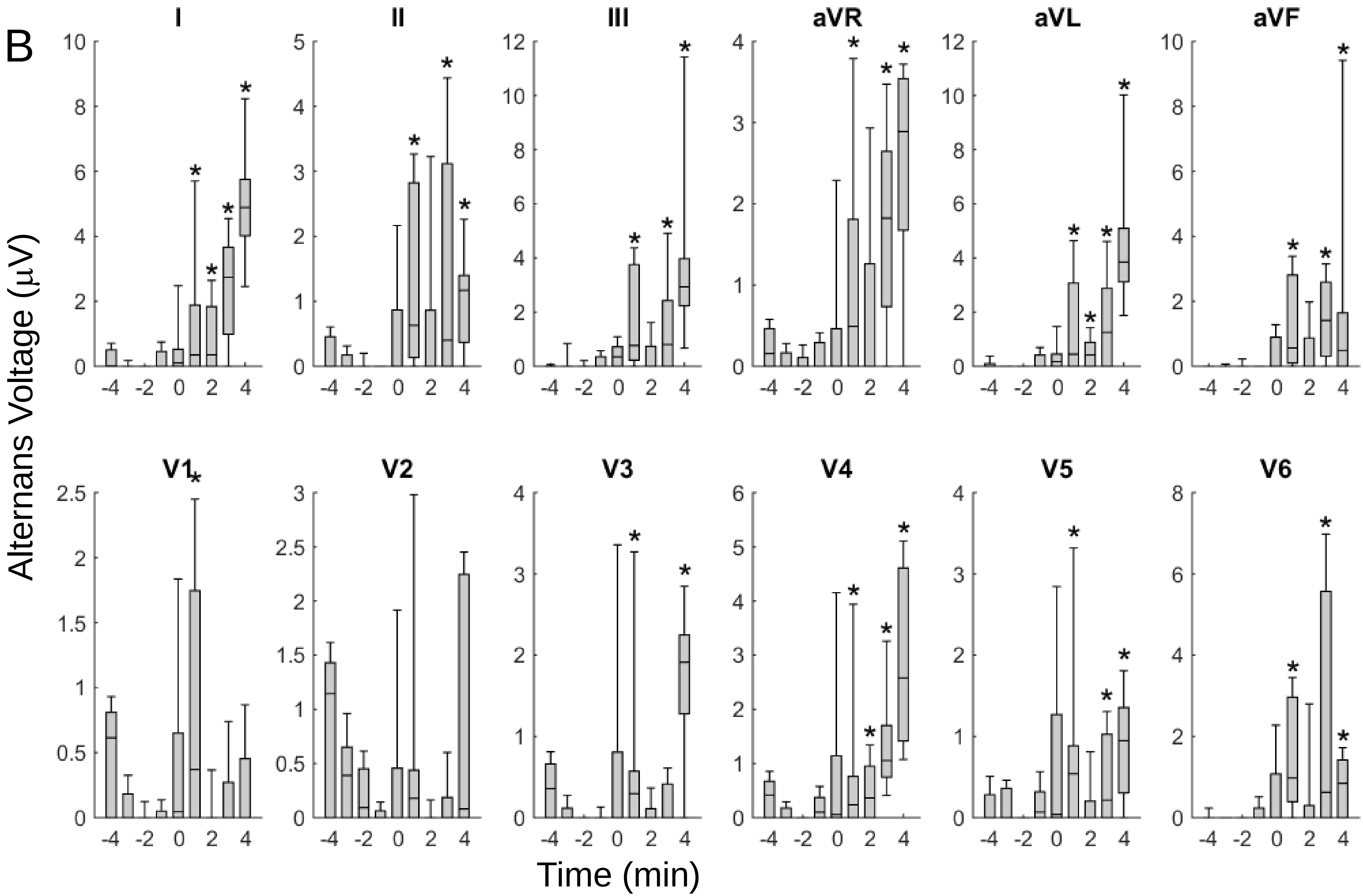

Online Supplement Figure 3

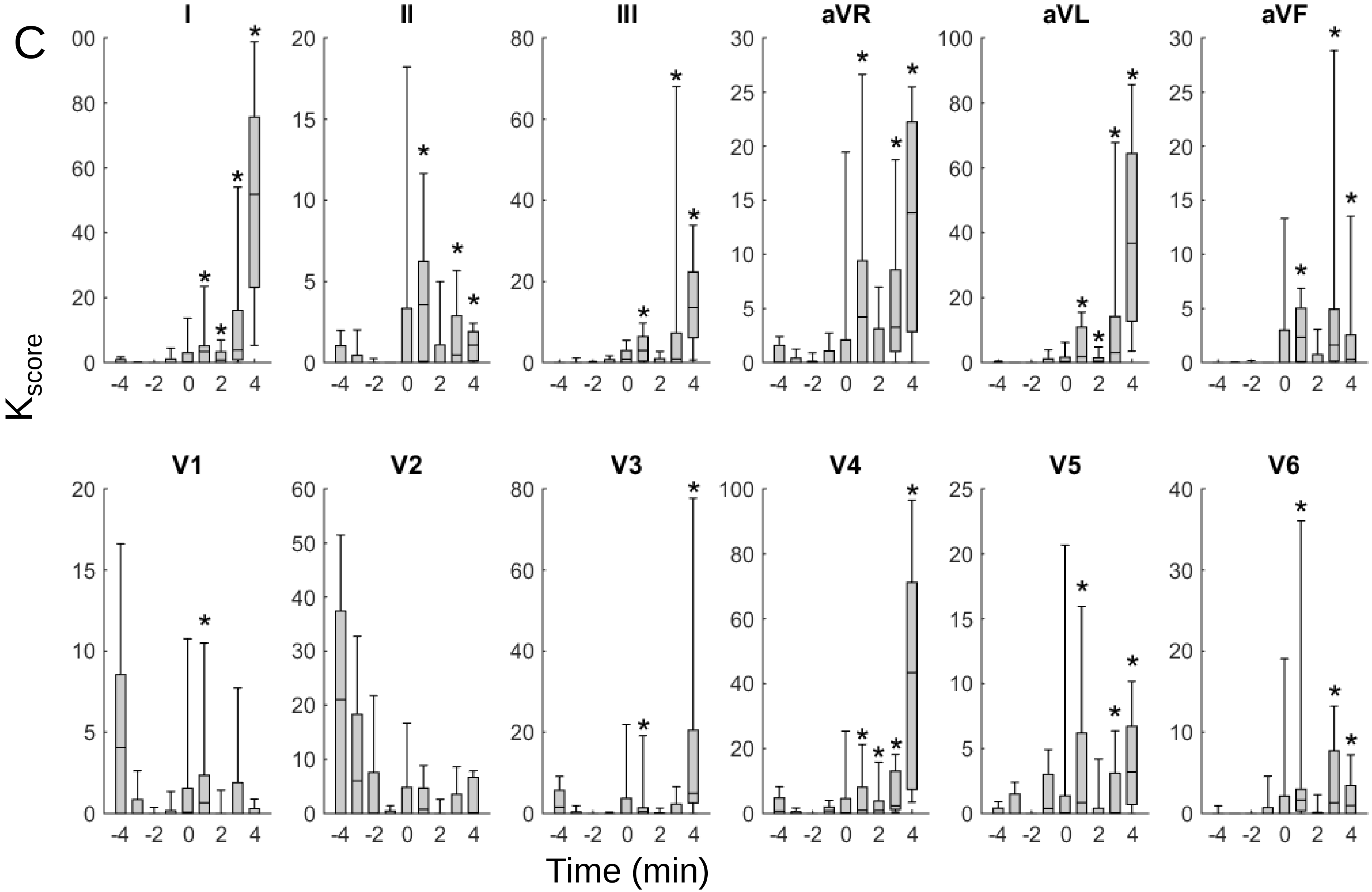

Online Supplement Figure 4

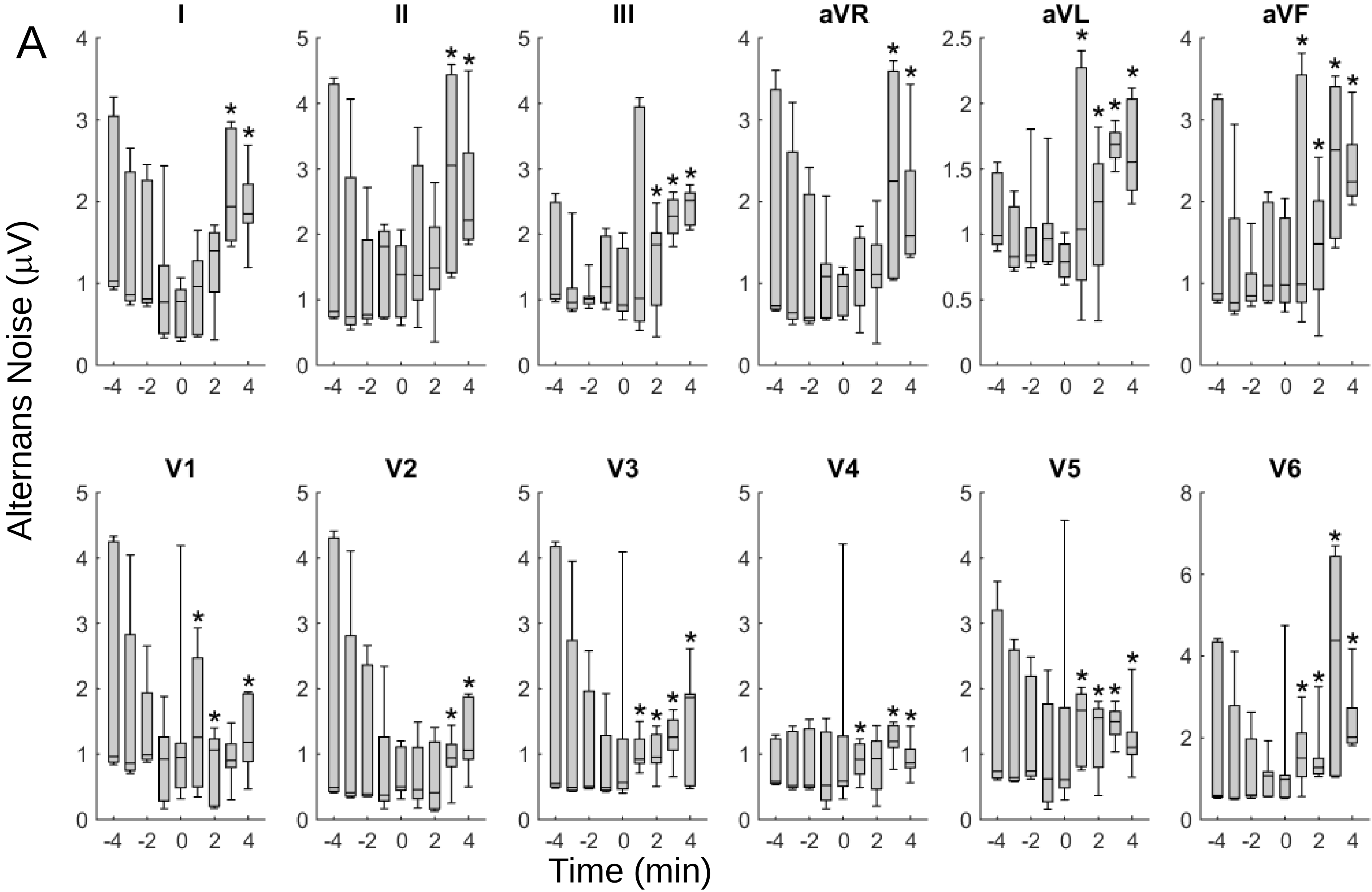

Online Supplement Figure 4

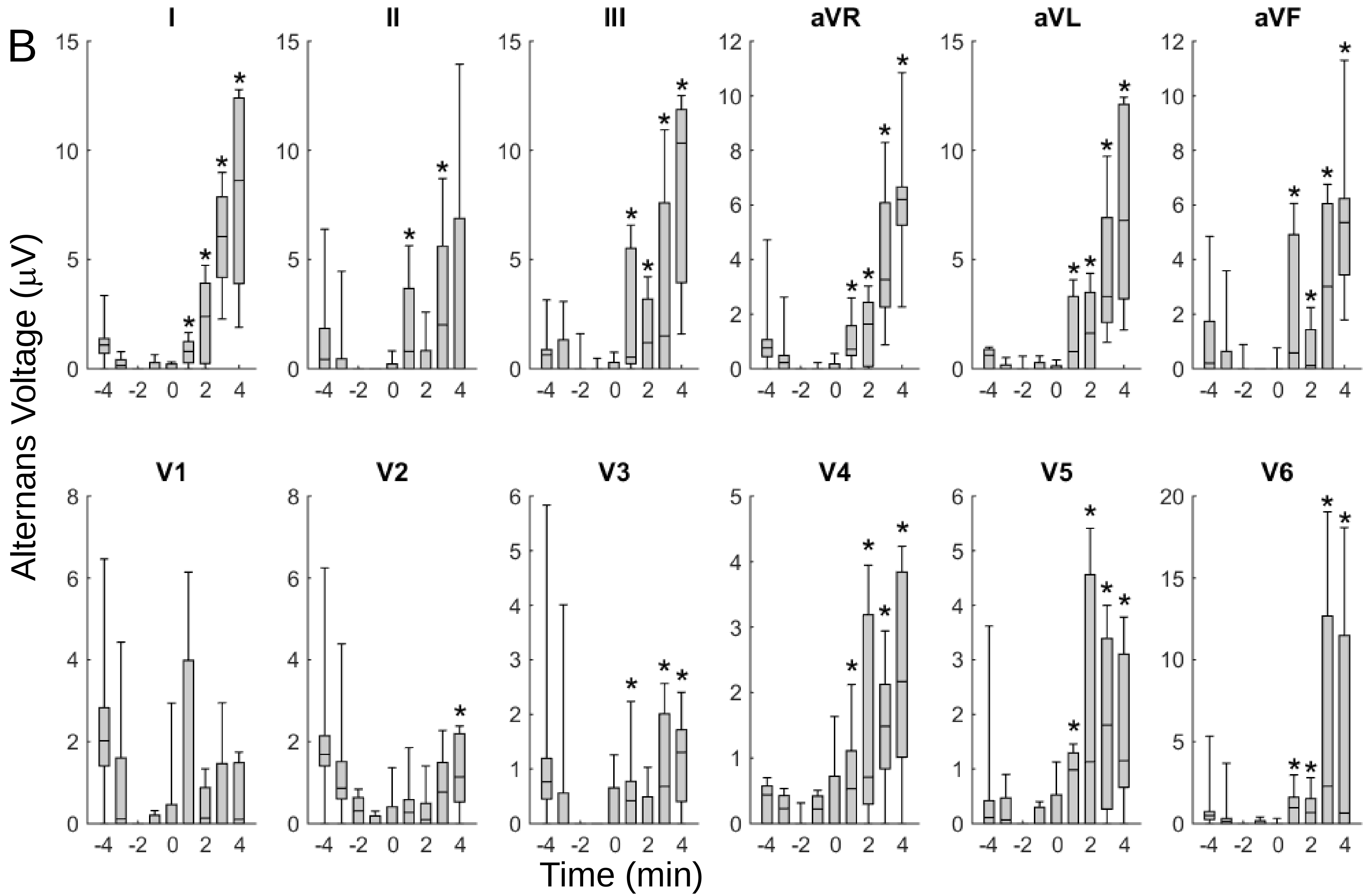

Online Supplement Figure 4

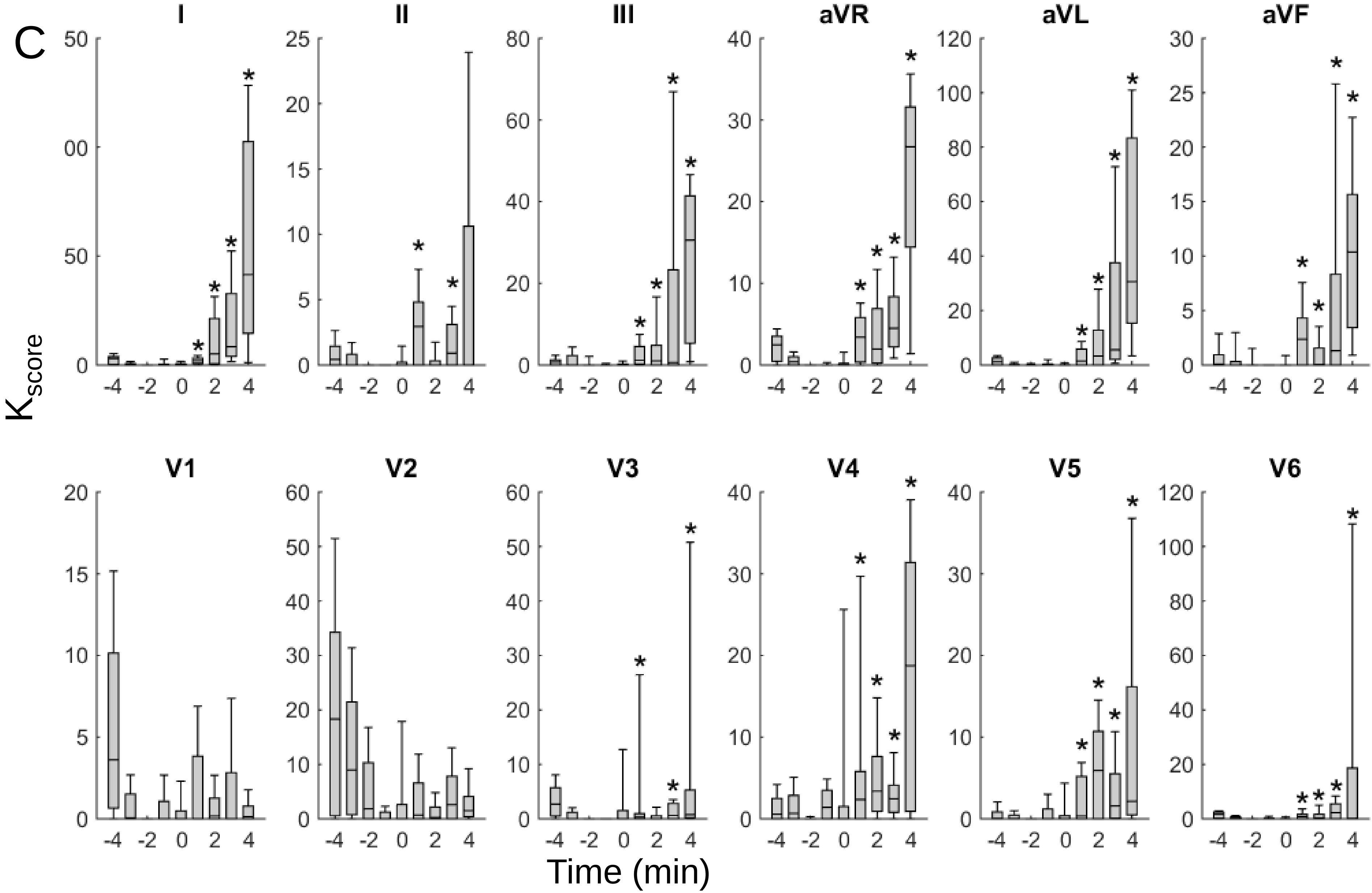

Online Supplement Figure 5

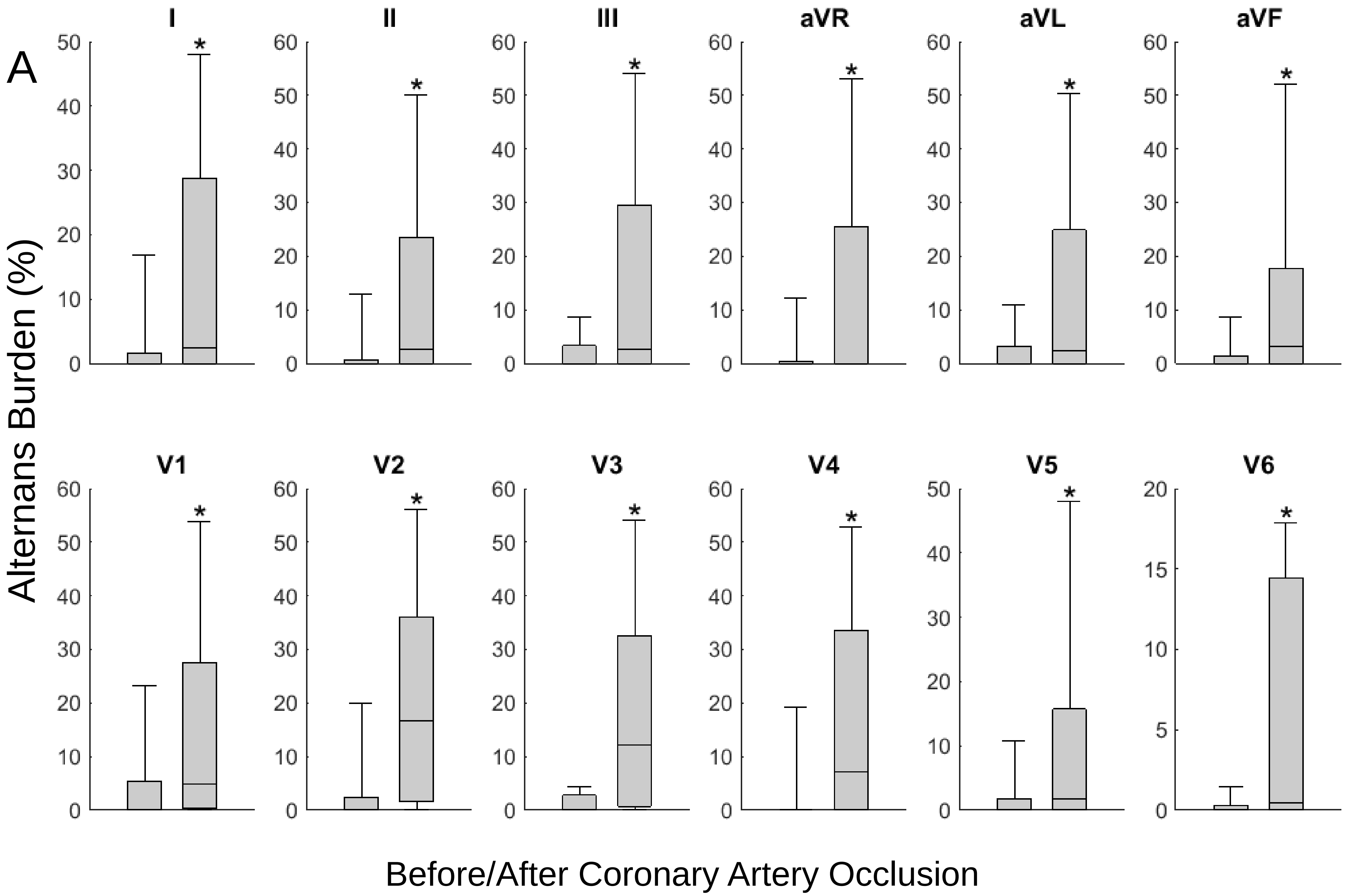

Online Supplement Figure 5

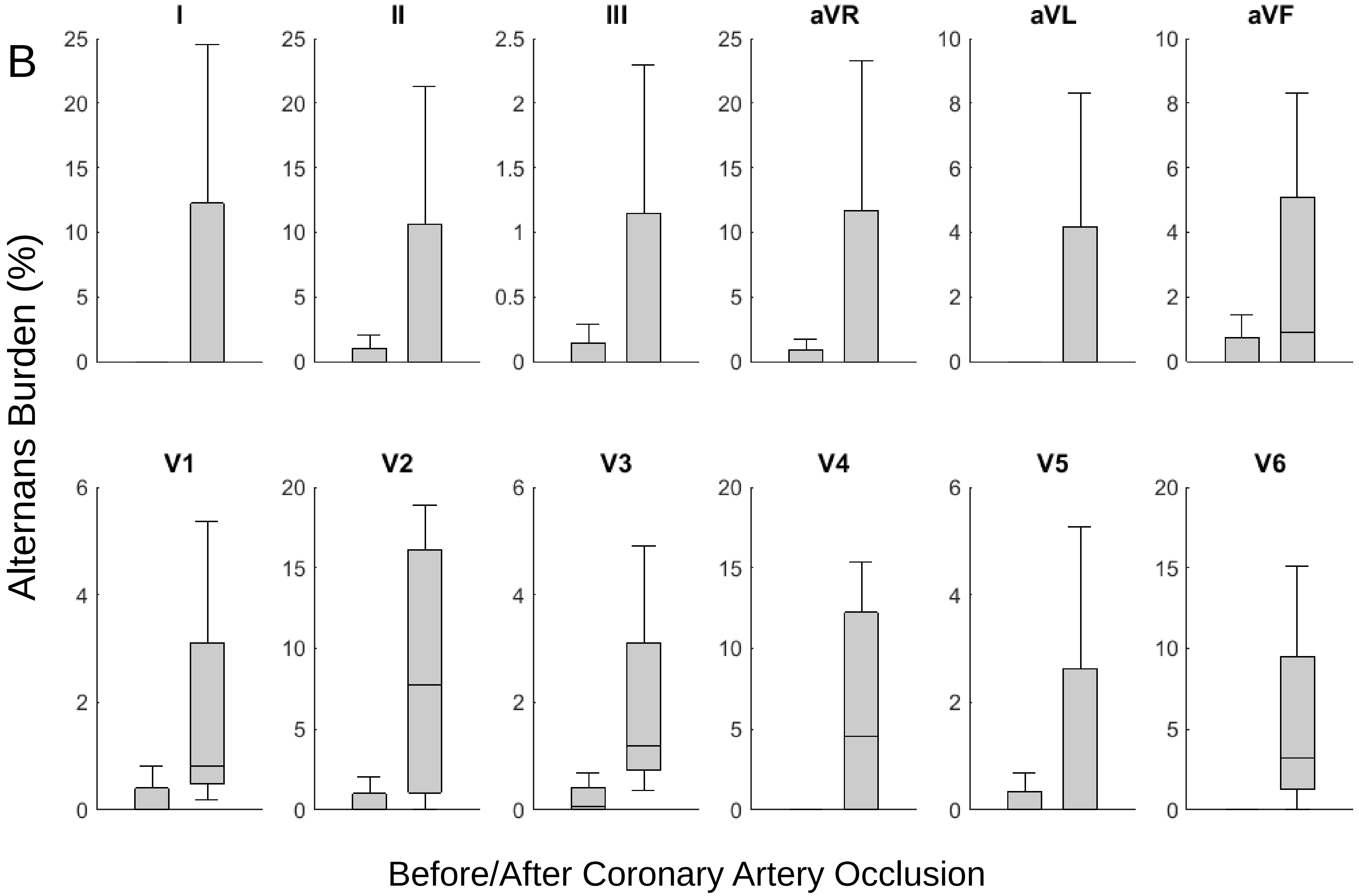

Online Supplement Figure 6

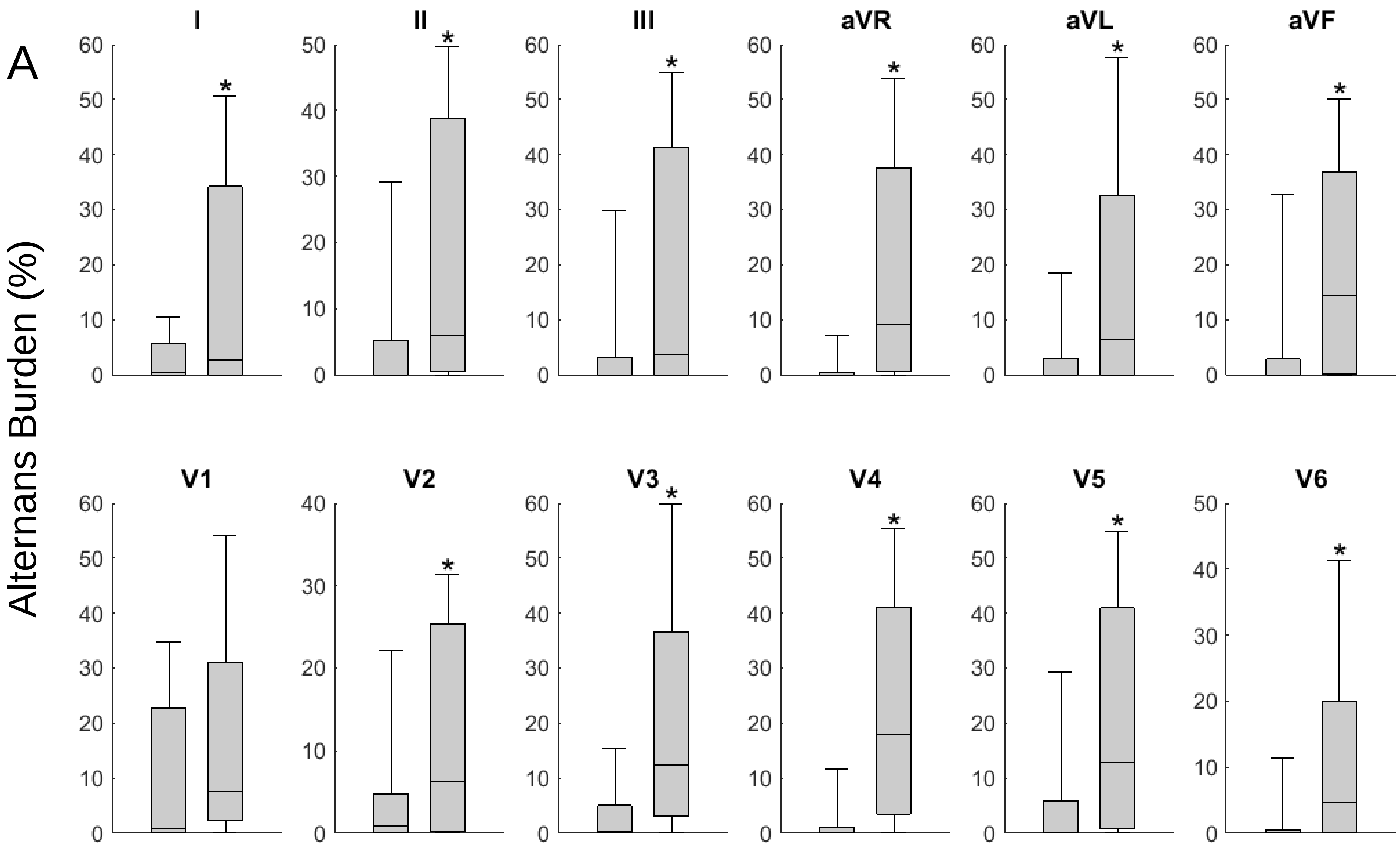

Online Supplement Figure 6

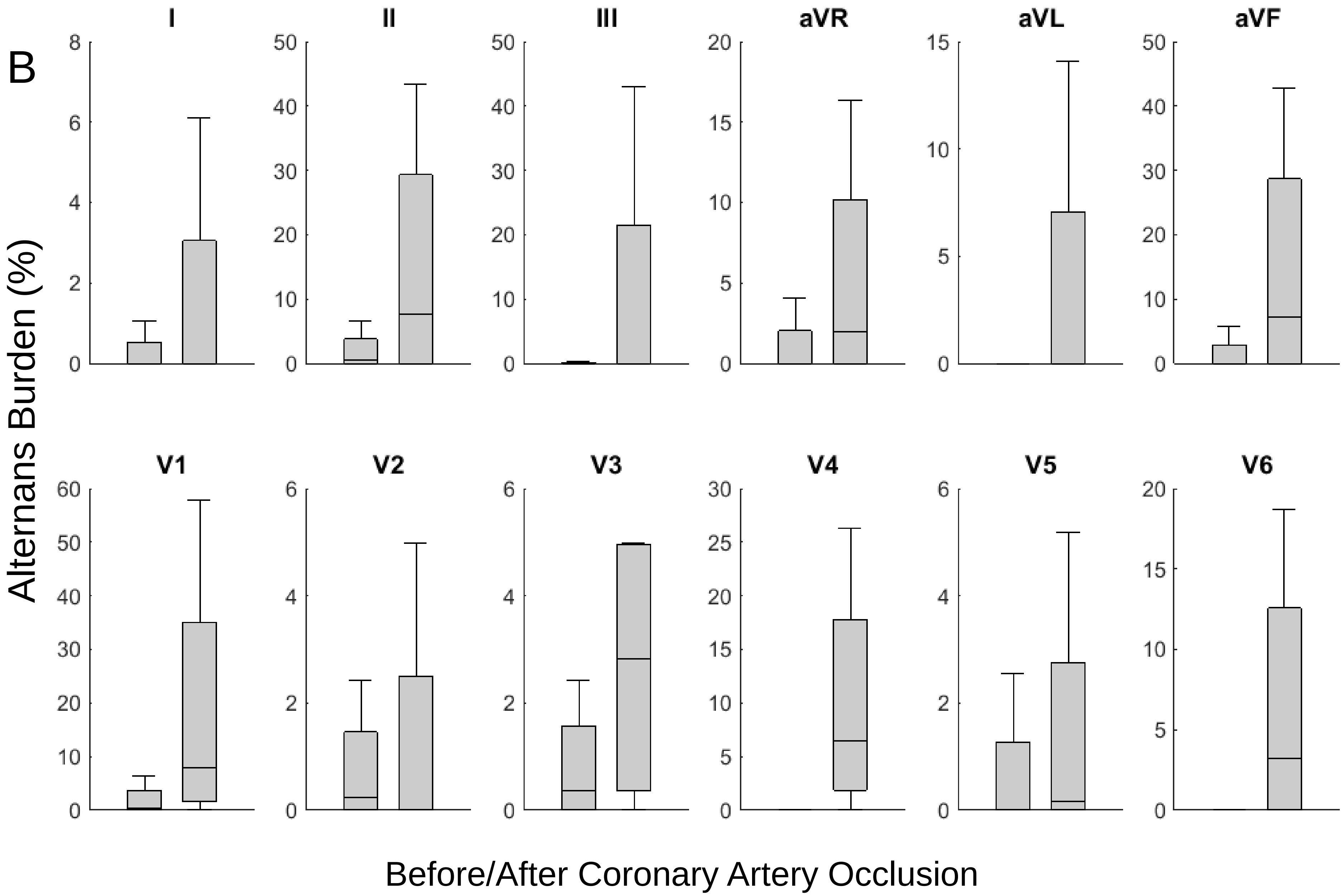

Online Supplement Figure 7

A

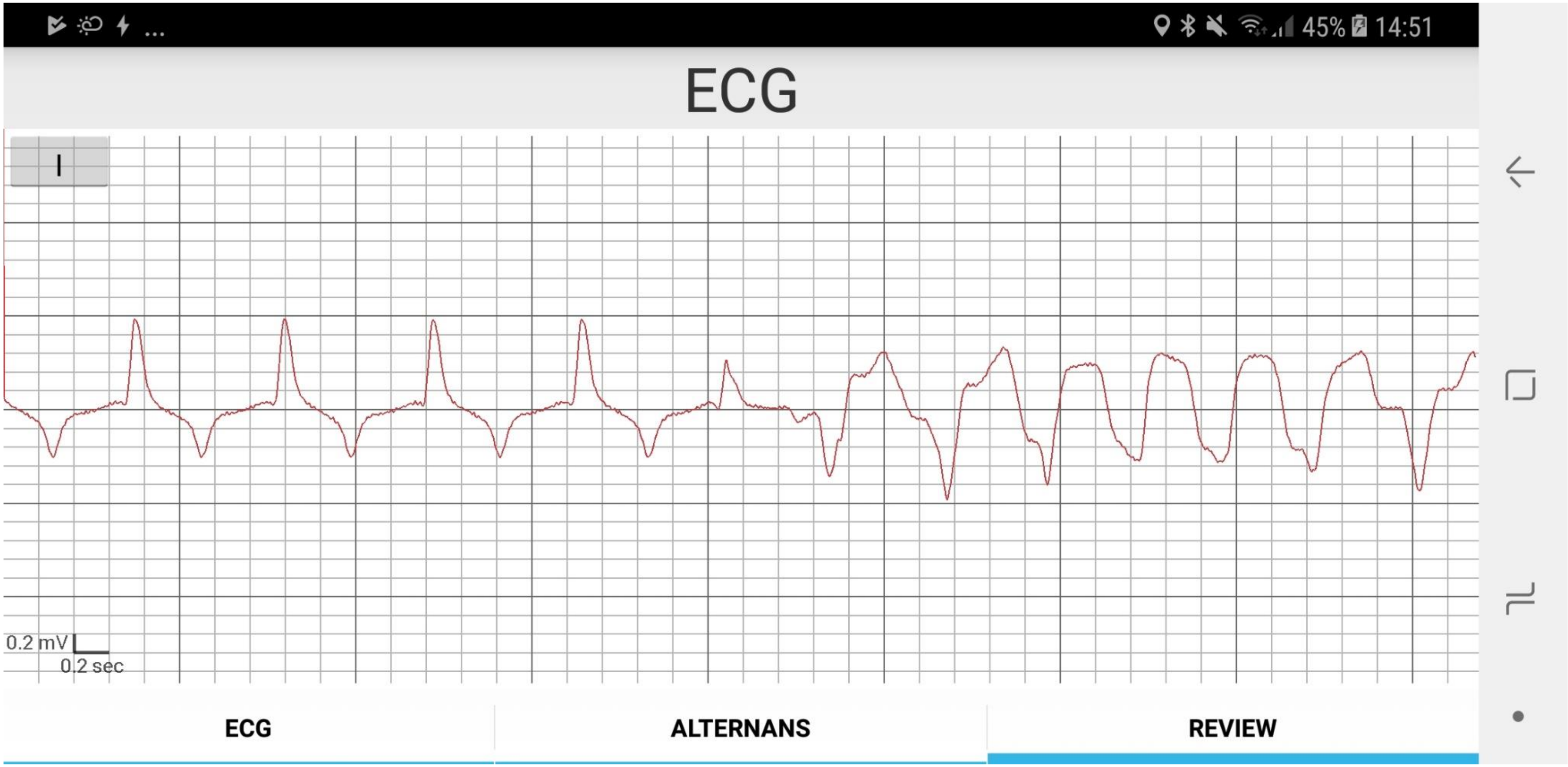

Online Supplement Figure 7

B

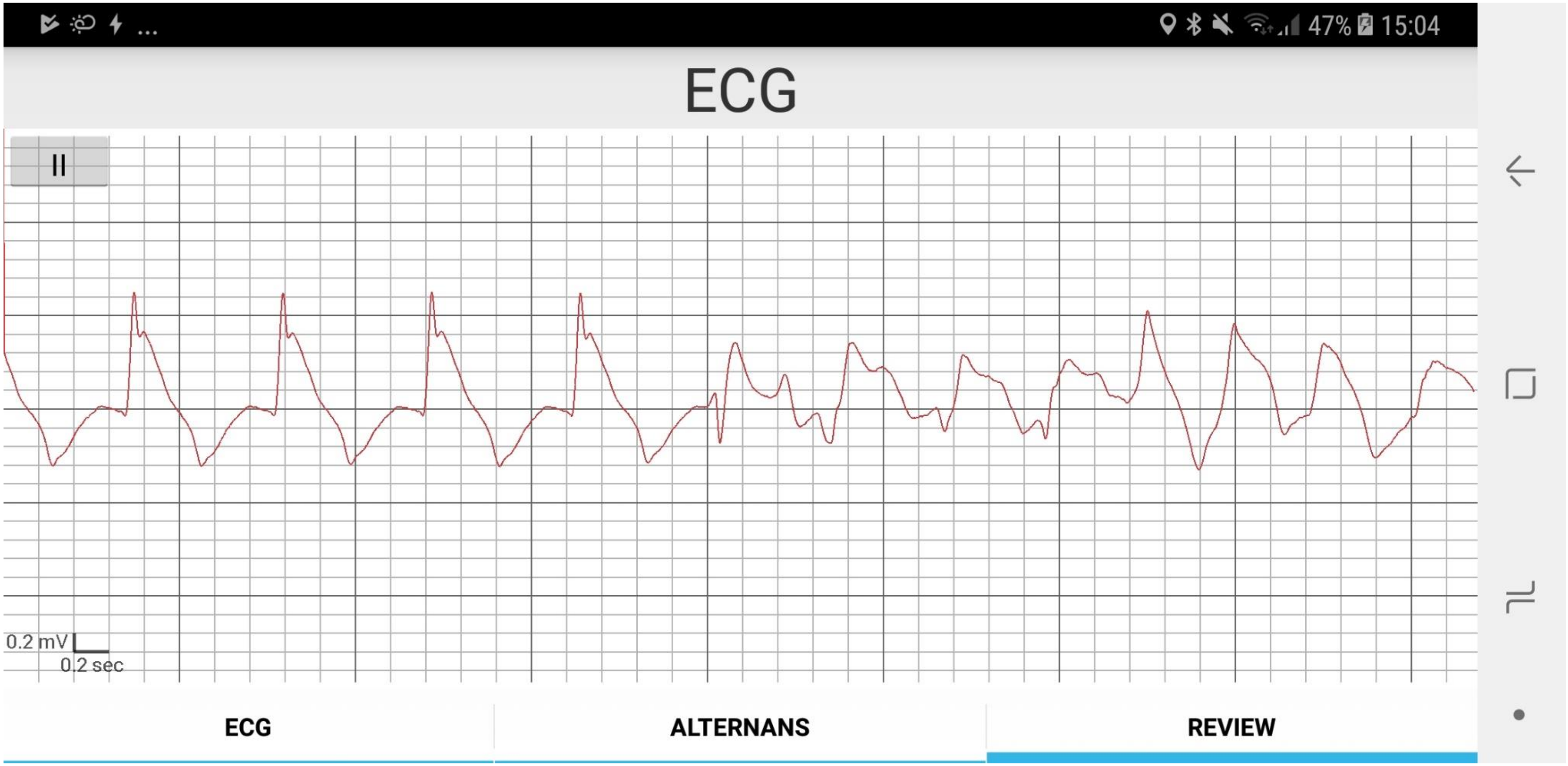

Online Supplement Figure 7

C

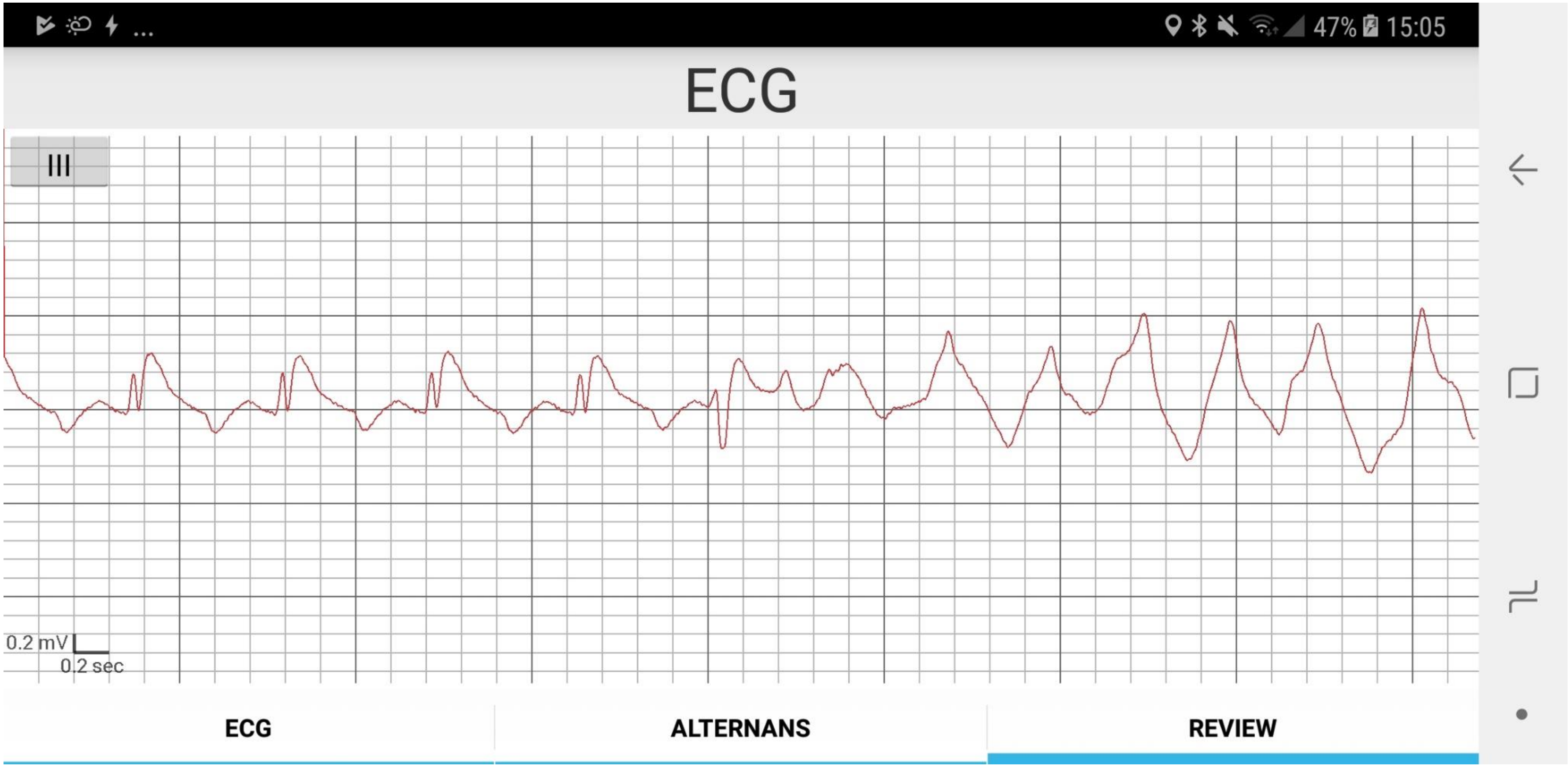

Online Supplement Figure 7

D

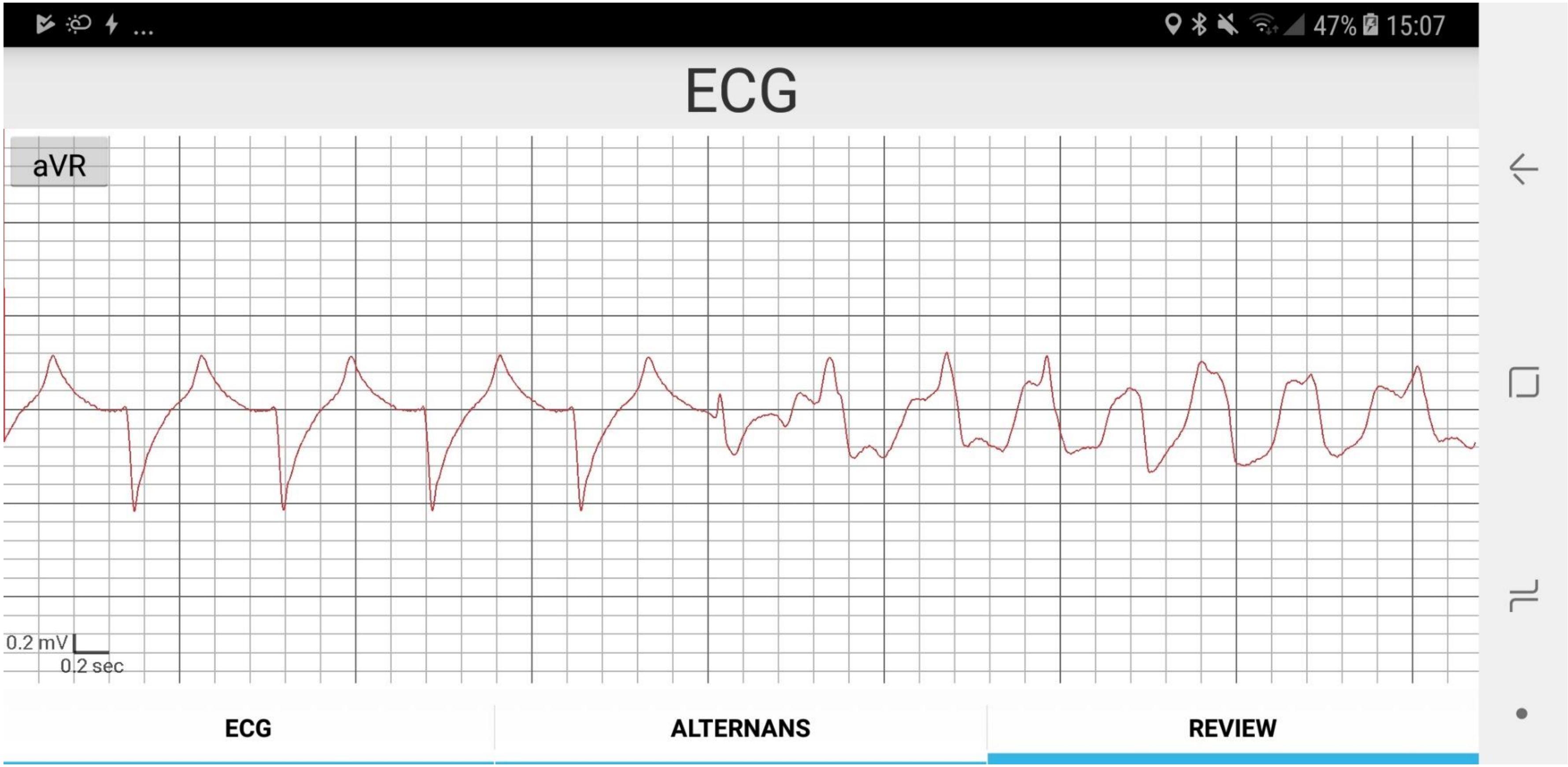

Online Supplement Figure 7

E

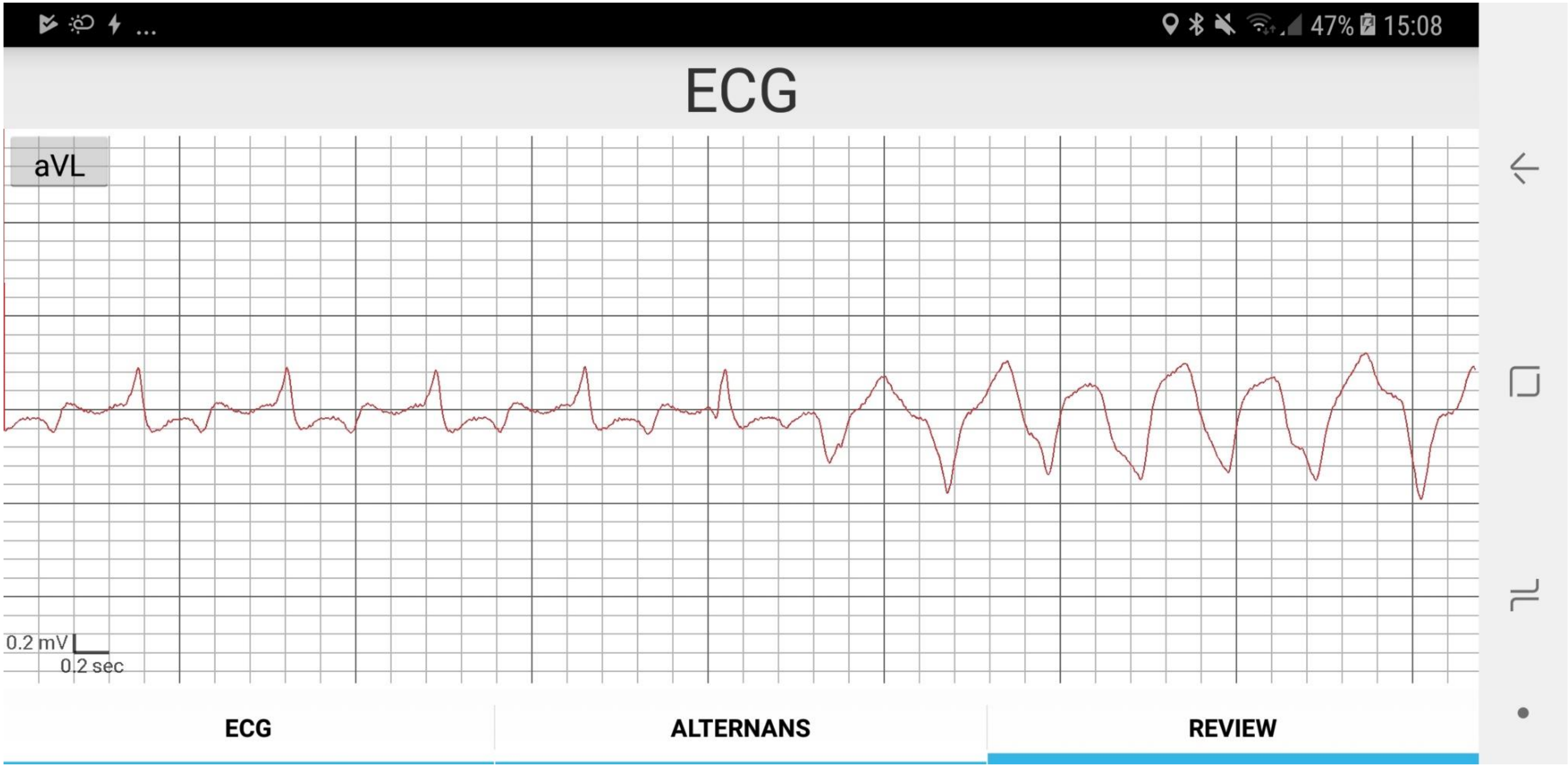

Online Supplement Figure 7

F

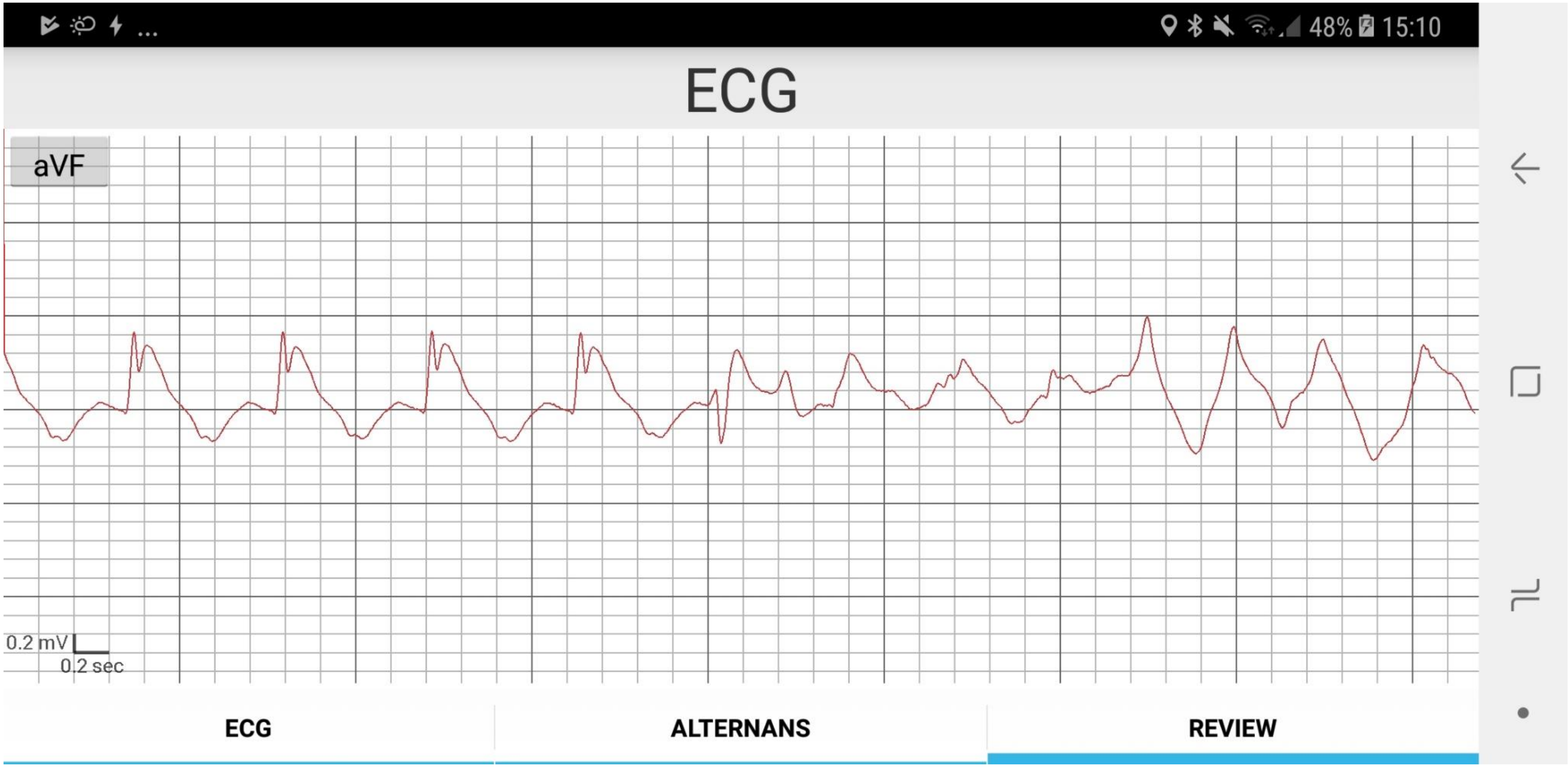

Online Supplement Figure 7

G

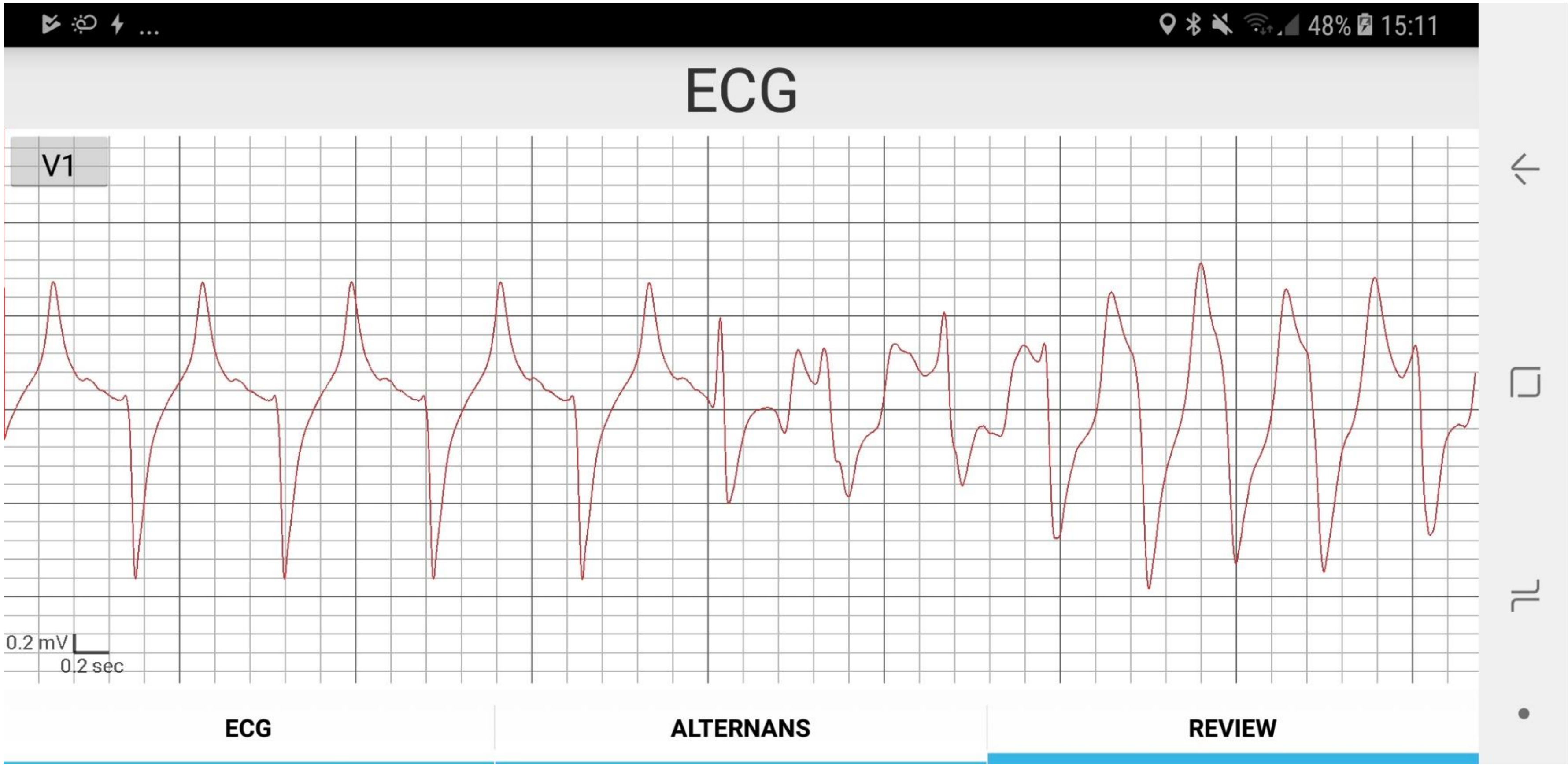

Online Supplement Figure 7

H

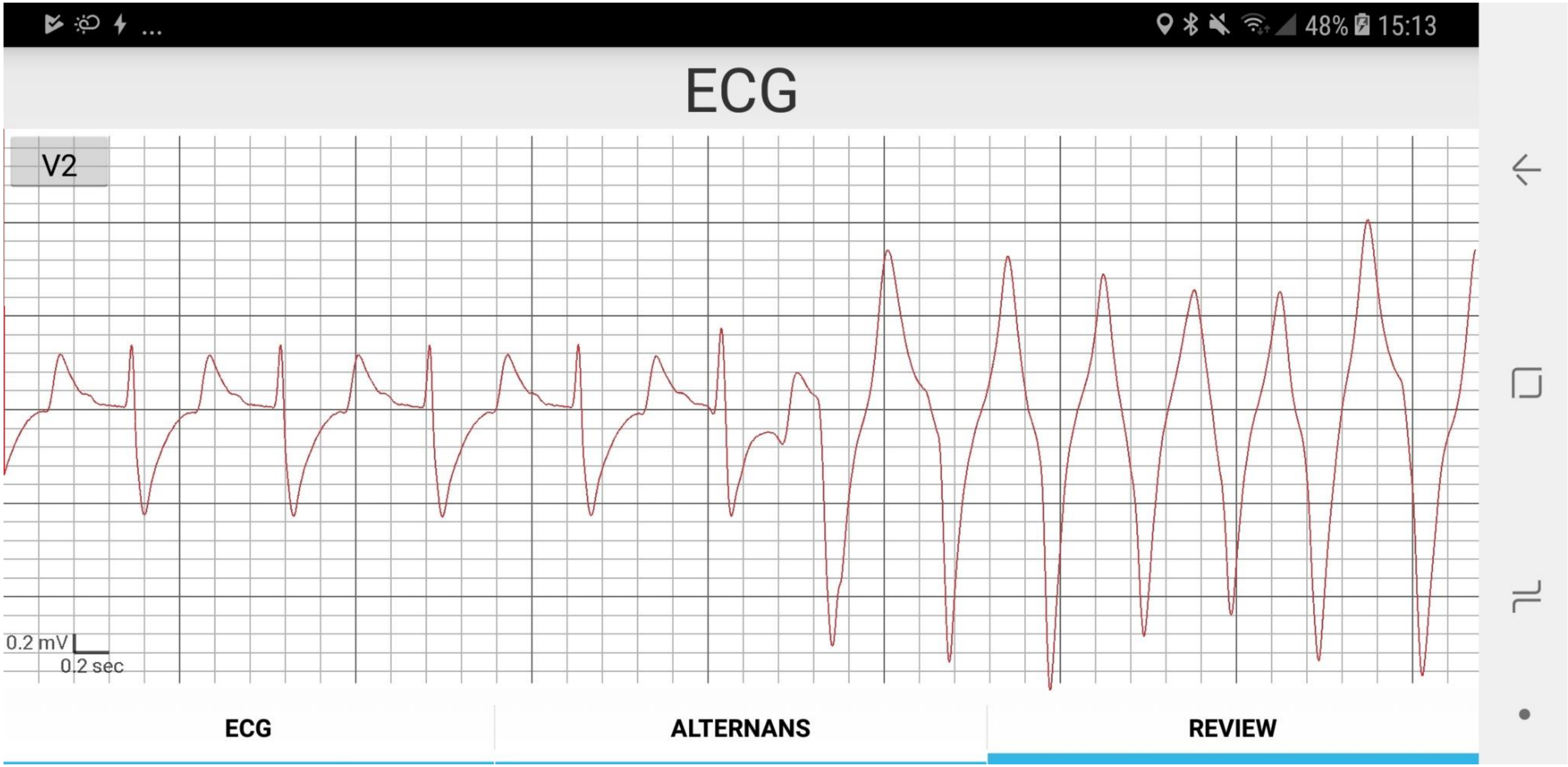

Online Supplement Figure 7

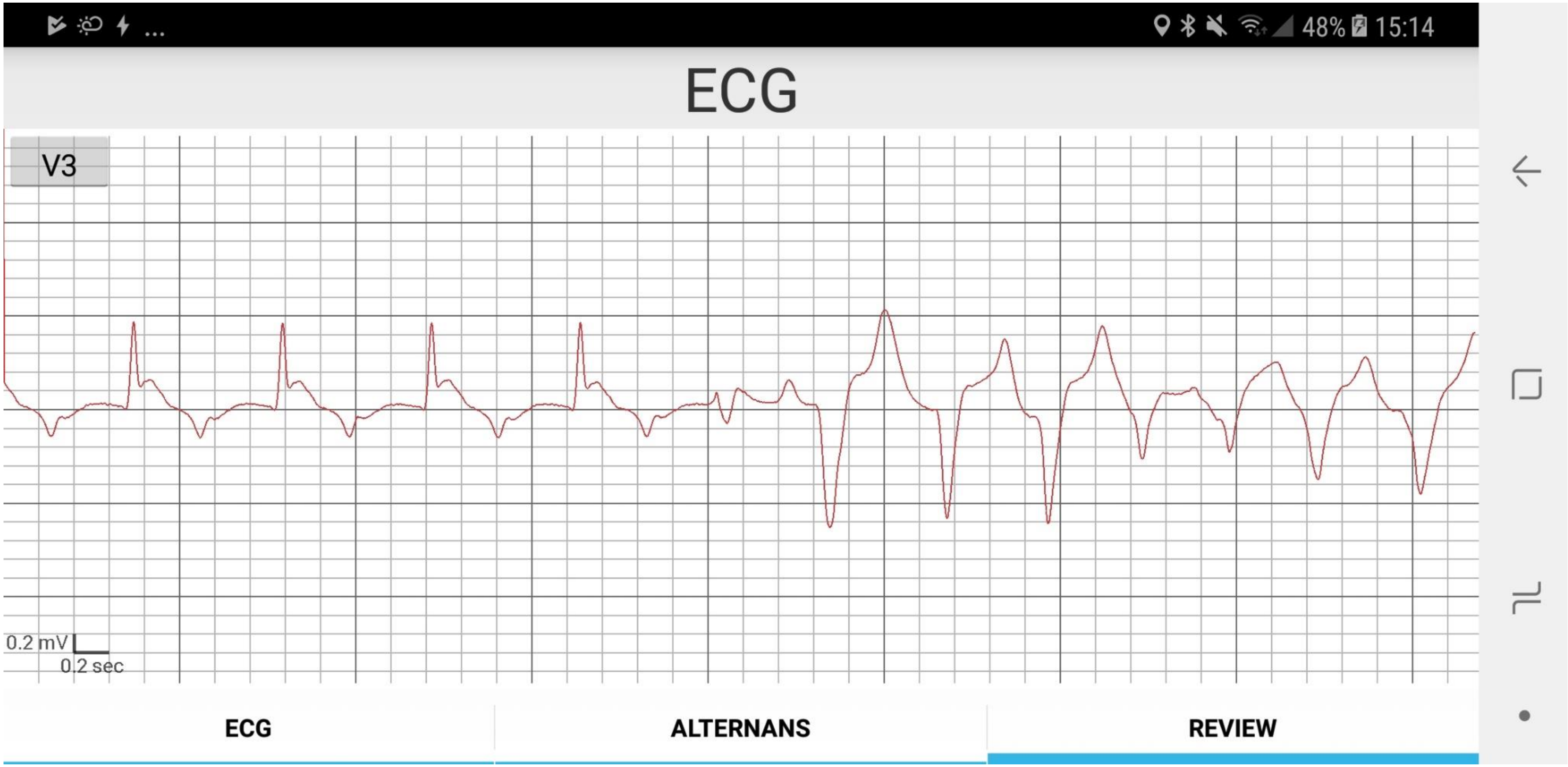

Online Support Figure 7

J

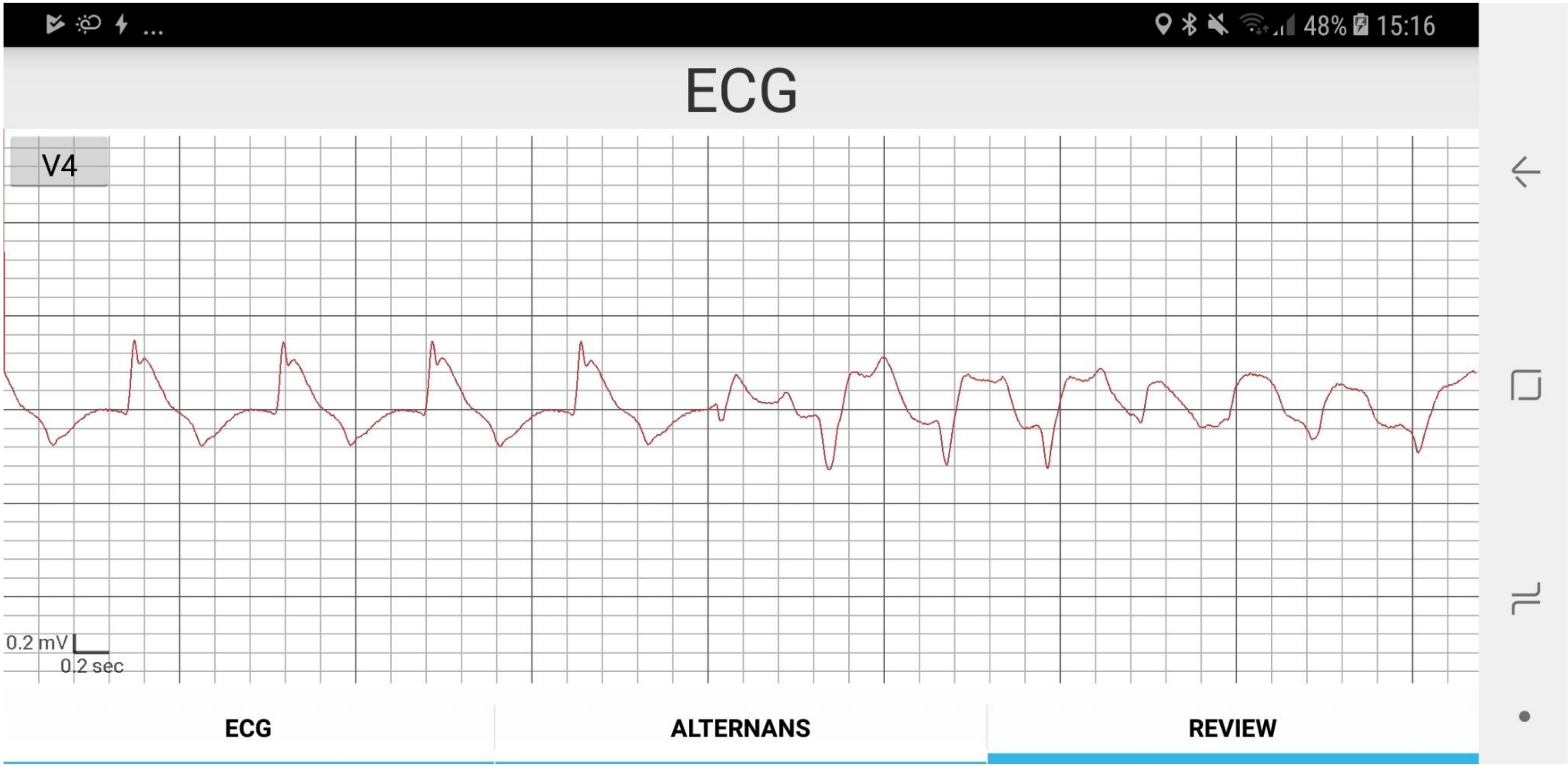

Online Support Figure 7

K

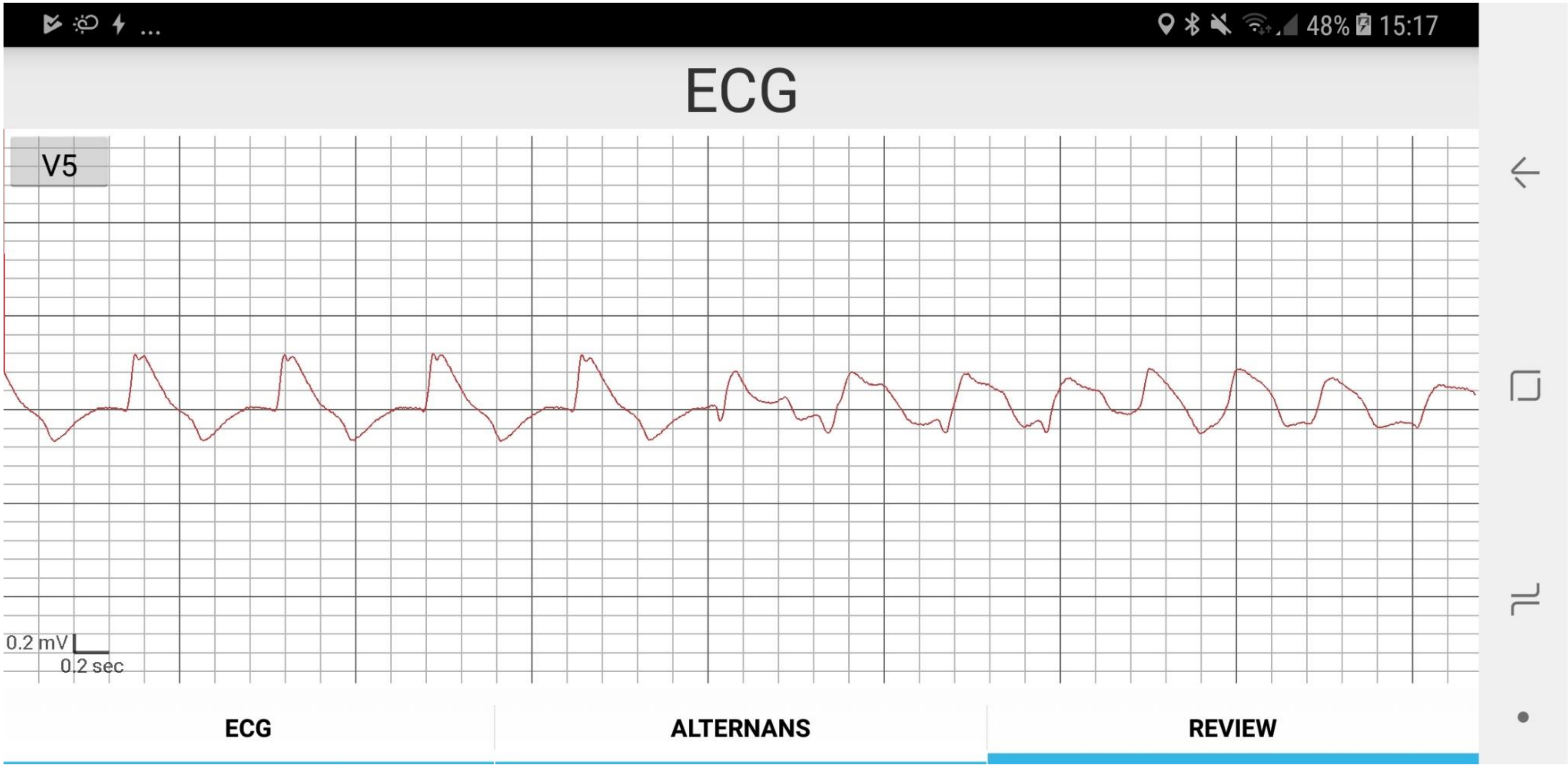

Online Supplement Figure 7

L

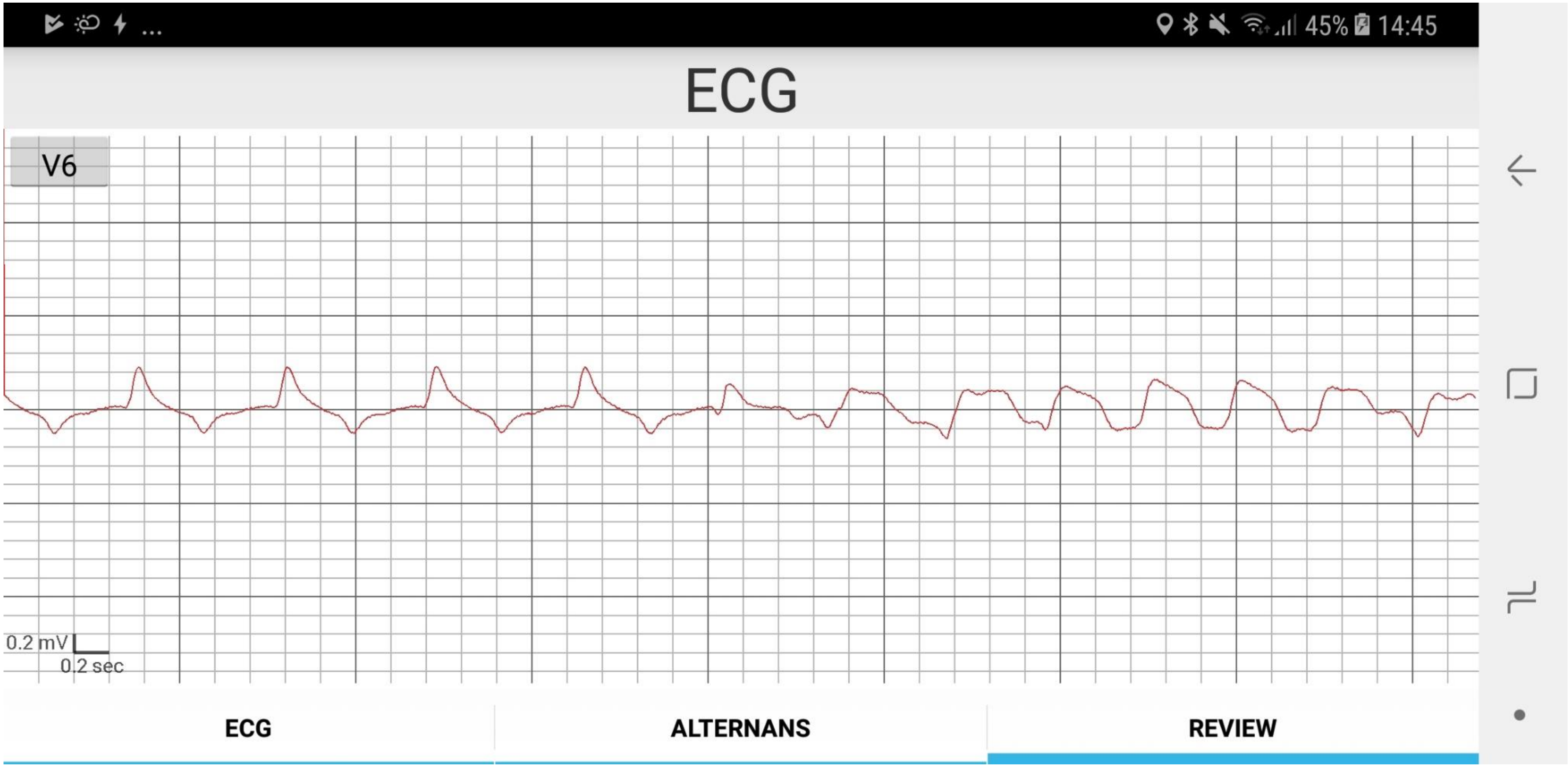

Supplement: Supplementary file 1 — Supplementary Information [file 41598_2019_50487_MOESM1_ESM.pdf]
